# How cognitive and environmental constraints influence the reliability of simulated animats in groups

**DOI:** 10.1371/journal.pone.0228879

**Published:** 2020-02-07

**Authors:** Dominik Fischer, Sanaz Mostaghim, Larissa Albantakis

**Affiliations:** 1 School of Management, Technical University of Munich, Munich, Germany; 2 Faculty of Computer Science, Otto von Guericke University of Magdeburg, Magdeburg, Germany; 3 Department of Psychiatry, Wisconsin Institute for Sleep and Consciousness, University of Wisconsin–Madison, Madison, Wisconsin, United States of America; Wroclaw University of Science and Technology, POLAND

## Abstract

Evolving in groups can either enhance or reduce an individual’s task performance. Still, we know little about the factors underlying group performance, which may be reduced to three major dimensions: (a) the individual’s ability to perform a task, (b) the dependency on environmental conditions, and (c) the perception of, and the reaction to, other group members. In our research, we investigated how these dimensions interrelate in simulated evolution experiments using adaptive agents equipped with Markov brains (“animats”). We evolved the animats to perform a spatial-navigation task under various evolutionary setups. The last generation of each evolution simulation was tested across modified conditions to evaluate and compare the animats’ reliability when faced with change. Moreover, the complexity of the evolved Markov brains was assessed based on measures of information integration. We found that, under the right conditions, specialized animats could be as reliable as animats already evolved for the modified tasks, and that reliability across varying group sizes correlated with evolved fitness in most tested evolutionary setups. Our results moreover suggest that balancing the number of individuals in a group may lead to higher reliability but also lower individual performance. Besides, high brain complexity was associated with balanced group sizes and, thus, high reliability under limited sensory capacity. However, additional sensors allowed for even higher reliability across modified environments without a need for complex, integrated Markov brains. Despite complex dependencies between the individual, the group, and the environment, our computational approach provides a way to study reliability in group behavior under controlled conditions. In all, our study revealed that balancing the group size and individual cognitive abilities prevents over-specialization and can help to evolve better reliability under unknown environmental situations.

## Introduction

*Intelligence is the ability to adapt to changes*. According to this prevalent perspective, possessing general intelligence [1,2] not only enables one to perform a task correctly under already known conditions, but also to perform well under unexpected conditions. Further, in natural environments intelligent behavior is not only dependent on the (maybe limited) intelligence of the individual organism, but also involves interactions with the social and physical environment [3–5]. The ability to adapt one’s behavior to the behavior of other group members is necessary to act appropriately in case of unforeseen events, not only in the animal world but also in *high-reliability organizations* (e.g., aircraft carrier or nuclear power plants) [6–8]–In the following, we use the term “reliability” to denote the ability of an organism to perform well even under slightly modified, unfamiliar circumstances.

While it seems intuitive that there is a triangular relationship between the individual, the group, and the environment [9], we discovered a lack of research on how individual behavior and group behavior are interrelated and depend on spatial attributes of the environment [10]. Several studies have investigated intelligence and knowledge on the group level, and some have modelled groups of individuals as single agents (e.g., [11–15]). These studies have their origins in a variety of disciplines and have in common that they seek to elucidate the dynamics between group members. However, our understanding of how an individual actor in a group evolves intelligent behavior and reliability is still limited.

Here, we are particularly interested in how an individual’s sensorimotor and memory capacity, the interaction between group members, and the environment constrain this evolution. To explore these factors in a controlled experimental setup, we used a simple evolution simulation, and we tested how specific cognitive and environmental limits influence the behavior, performance, and reliability of artificial organisms evolved in groups of various sizes.

Inspired and motivated by Pinter-Wollman et al. [10], we investigated how the behavior and performance of evolved “animats” (simulated agents with cognitive abilities [16,17]) varies in different task conditions, such as changes in the proportions of static objects, dynamic objects (moving group members), and individual sensorimotor and memory architecture. Using a simulation approach enabled us to manipulate and observe three dimensions which might influence evolved task performance and reliability: the group size (influencing the density of animats present in the environment), the animats’ architecture (that is, the maximal number of available sensors, motors, and memory units), and the environmental design. In this study, we explicitly distinguish between the final task performance reached in the evolution environment (“evolved fitness” (EF)) and the post-evolutionary “task fitness” (TF), which measures the performance of the evolved animats under specific modified conditions (not encountered during evolution). High task fitness across many modified conditions indicates high reliability. High evolved fitness, but low reliability could then be interpreted as a form of narrow intelligence, while high evolved fitness and high reliability would point to more general intelligence.

**Fig 1:**
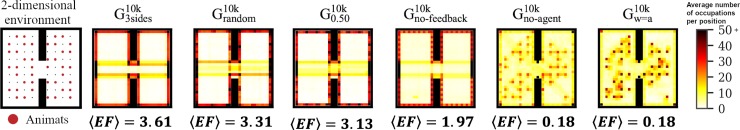
The average number of occupations per position in the final generations. The first panel on the left shows the two-dimensional environment, including two rooms with a total of 72 start positions (32 black dots [not occupied], 32 red dots [occupied]) for reference. In each trial, a subset of position is randomly selected as the animats’ initial locations. The other six panels show the average number of occupations per position as heat maps. The average is taken across time (500 time steps) and evolution simulations (30 per evolutionary setup). Red fields indicate high occupancy, and yellow fields indicate low occupancy in the corresponding position throughout the trial. Generally, well-performing animat groups evolve a wall-following strategy. 〈EF〉 indicates the mean evolved fitness of the final generation in the specific condition (see Results section for formal definition).

We used a genetic algorithm to let the animats’ behavior evolve under various evolutionary setups. Specifically, the animats were controlled by *Markov brains (MBs)* [17], which consisted of computational units whose functions and connectivity were determined by the animats’ adaptive genome. The animats’ task was to navigate through a two-dimensional world composed of two rooms without colliding with other group members (see Fig 1). Each animat could achieve a maximum score of ***4*** *points* within each trial, with a small penalty (**-*0*.*075*** *points*) for each collision and a large reward (***+1*.*0*** *points*) for crossing gates between rooms. After an evolution of 10,000 generations, we tested the final animats under modified task conditions modeled as: a variation in group size (the number of animats simultaneously present in the environment), the complexity of the static obstacles in the environment, and interaction rules between animats that affect task difficulty. The interaction rules include changes in the animats’ ability to differentiate between static obstacles and other animats, the imposed collision penalty, and the possibility to inhabit the same location in the environment. An animat was considered reliable if its task performance remained high across many variations of these test conditions.

A predecessor study focused on the influence of group size on the evolution of group fitness and reliability [18], while the present work (1) extends the reliability experiments, (2) includes evolutionary setups with variations in the animats’ architecture, and (3) elaborates the measurement of brain complexity by applying measures developed within the framework of the integrated information theory (IIT) to the evolved MBs [19,20]. There are two additional works which directly relate to our study: First, Konig et al. [21] provided the original experimental setup. They designed a two-dimensional spatial-navigation task in which a swarm of robots has to learn to travel between two rooms. Second, Albantakis et al. [20] showed how single animats evolve in a perceptual-categorization task environment with dynamic objects under various task difficulties. The primary motivation behind their work was to investigate the evolution of integrated information [19], which is an indicator for brain complexity, and its relation to task difficulty and memory capacity. Here, we discuss how the complexity of the MBs—evolved in the various experimental setups—is related to reliability as a prerequisite for general intelligence.

Overall, we found that, specialized animats can be reliable under the right conditions, that feedback from the motor units has an impact on performance and reliability, that animats benefit from passive interaction, and that more sensors enable reliability with simpler and less integrated brain structures (which challenges the view that higher generalized intelligence is necessarily associated with more complex cognitive architectures). Generally, our approach highlights the complexity of the dependencies between the three investigated dimensions: properties of the individual, group interaction, and environmental design. Even the simplified conditions of our simulation experiments make this complexity visible, and thus cautions against hasty generalizations, e.g., across different species or environments.

In the following, we will first present our results on the animats’ task performance, reliability, behavior, and brain complexity across varying evolutionary setups. After that, we will discuss the findings in the broader scope of the literature and also how our work contributes to it. The last part of the work explains the methods and research design.

## Results

We simulated the evolution of artificial organisms (“animats”) with diverse cognitive architectures (number and type of available sensors, motors, and memory units) for 10,000 generations under various conditions. See Table 1 for an overview of all evolution simulations conducted.

**Table 1:** Definition of simulation conditions (“evolutionary setups”). Evolutionary setups are indicated by a label G_i_, where the index i specifies the respective type of evolutionary setup. Differences compared to baseline configuration (top row, ***G*_*0*.*50*_**, group size of 36 animats) are highlighted in bold.

Label	G_i_	Absolute Group Size^1^	Cognitive Architecture^2^	Interaction Condition^3^	Sensor Configuration^2^	Results in Figs
**Varying group size**	***1*.*00***^4^	72	4 memory units 2 motors with feedback	Active Penalty, blocking disabled	1 animat sensor, 1 wall sensor	2/3/4
	***0*.*75***	54				
	***0*.*50***	36				
	***0*.*25***	18				
	***single***	1				
	***random***	random				
**Varying cognitive architecture**	***bigbrain***	36	8 memory units	Active Penalty, blocking disabled	1 animat sensor, 1 wall sensor	5/6/7
	***smallbrain***		2 memory units			
	***no-feedback***		4 memory units 2 motors without feedback			
**Varying** **interaction conditions**	***no-penalty***	36	4 memory units 2 motors with feedback	No Penalty, blocking disabled	1 animat sensor, 1 wall sensor	8/9/10
	***blocked/*** ***no-penalty***			No penalty, blocking enabled		
	***blocked***			Active Penalty, blocking enabled		
**Varying sensor configuration**	***no-agent***	36	4 memory units 2 motors with feedback	Active Penalty, blocking disabled	1 wall sensor	11/12/13
	***3sides***				3 animat sensors, 3 wall sensors	
	***w = a***				1 universal sensor	

^1^ Absolute group size, 72 animats corresponds to 100% coverage of available starting slots.

^2^ See Methods section for detailed architecture. Numbers indicate maximally available sensors, motors, or memory units, not the actually evolved number, which may be less.

^3^ If penalty is active, animats receive penalty (-0.075 points) for colliding with other animats. If blocking is active, animats are not able to share the same position, otherwise they can occupy the same position, albeit with a penalty.

^4^ Numeric indices correspond to relative group size: 1.00 corresponds to 100% coverage of available starting slots (100% ≙ 72 animats). The indicators 0.75, 0.50, and 0.25 correspond to 75%, 50% and 25% of available starting slots, respectively.

All animats were evolved to travel between two rooms in a two-dimensional environment, which they shared with other animats of their same type (“clones” with the same genome), except in the “single” condition (see Fig 1(A) and Table 1). The evolutionary fitness selection occurs at the level of the genome (each generation consists of a population of 100 genomes) and is positively dependent on the average number of times that the corresponding animats (“phenotype”) stepped through the gate (***+1*.*0*** points) between the two rooms. After a successful gate crossing, the same animat did not receive another reward for 100 time steps to avoid crowding at the gate. In addition, we imposed a small penalty each time they collided with other animats (***-0*.*075*** points, if not stated otherwise). Throughout, fitness values are displayed as absolute numbers with a maximum value of ***4*** *points* (corresponding to the maximal number of possible gate crossings without collisions). A detailed description of the task environments and the *evolutionary algorithm* is provided below in the Methods section.

In many evolutionary setups (Table 1), high final fitness values (***EF > 3***, *“evolved fitness”*) were reached. Fig 1(B) displays six different heatmaps visualizing several evolved movement patterns. It is observable that animat groups with reasonable evolved fitness (***EF***) converge towards a “swarm”-like wall-following behavior, which is determined by both, interactions with fellow animats and interactions with the environment [4,10].

Once evolved, the best genome of each final generation was selected for post-evolutionary tests under modified conditions. Specifically, we modified the following three environmental factors: (1) the number of co-existing animats, (2) the complexity of static obstacles compared to the original two-dimensional environment (see Fig 1(A), and the Methods section for details on the environmental design), and (3) the interaction conditions between agents (see Table 2). For each test condition we assessed the “task fitness” (***TF***) achieved in the particular post-evolutionary test environment (to be distinguished from the animats’ evolved fitness (***EF***) reached after 10,000 generations in its original evolutionary setup). In addition, we evaluated the animats’ behavior and quantified their reliability (average task fitness across modified conditions) across varying group sizes in the original environment (***R***).

**Table 2:** Overview of the eight environments in which reliability tests were performed. They differ in environmental conditions and in the complexity of the world design.

Label	Environmental Conditions	Environment (see Methods)
Original	Active penalty^1^, blocking disabled^2^	See Fig 16(A)
No Penalty	No penalty, blocking disabled	See Fig 16(A)
Blocked	Active penalty, blocking enabled	
Blocked and no Penalty	No penalty, blocking enabled	
Noisy Corners	Active penalty, blocking disabled	See Fig 16(B)
Small Gates		See Fig 16(C)
4 Rooms		See Fig 16(D)
4 Messy Rooms		See Fig 16(E)

^1^ If penalty is active, animats receive penalty (-0,075) when colliding into each other.

^2^ If blocking is active, an animat cannot move onto the location of another animat.

Finally, we quantified the complexity of the evolved MBs using two measures developed within the framework of integrated information theory (IIT) [19,20]: the *integrated information* (***Φ***^***Max***^) and the corresponding *number of concepts* (***#Concepts(Φ***^***Max***^***))*. *T***he analysis was performed using “PyPhi”, the IIT Python toolbox [22], using the standard settings according to [19]. PyPhi takes the evolved MBs as an input in form of their “transition probability matrix” (TPM). The TPM specifies how the states of the MB’s computational units (e.g., motors and memory units) update, given the state of their inputs. In this study, all computational units are binary and deterministic (see Methods “Animat Architecture”). Briefly, ***Φ q***uantifies how much of the information specified by all components of a system would be lost under a partition of the system. ***Φ*** has been proposed as a measure of complexity, as it will be high for systems with many different components (functional differentiation) that are also highly integrated [19,23]. For a particular MB we identify the subset of computational units with the maximal amount of integrated information as ***Φ***^***Max***^. For this subset, we also measure the number of components (“concepts”) ***#Concepts(Φ***^***Max***^***)*.** A “concept” in IIT is a subsystem that has a causal role within the system—a mechanism within the system. A concept causally constraints both, the past and future states of the system, and is irreducible to its parts. ***#Concepts(Φ***^***Max***^***)*** thus captures the number of internal functions performed by the subsystem with ***Φ***^***Max***^. For details please refer to the original publication [19] and to [20] for an application of these measures to evolved MBs. While there may be simpler, less computationally demanding options for evaluating the causal complexity of the evolved MBs (see [16,17,24]), the chosen measures are fairly well established [20,22,23,25] and are theoretically motivated as part of the formal framework of the integrated information theory (IIT) [19].

**Fig 2:**
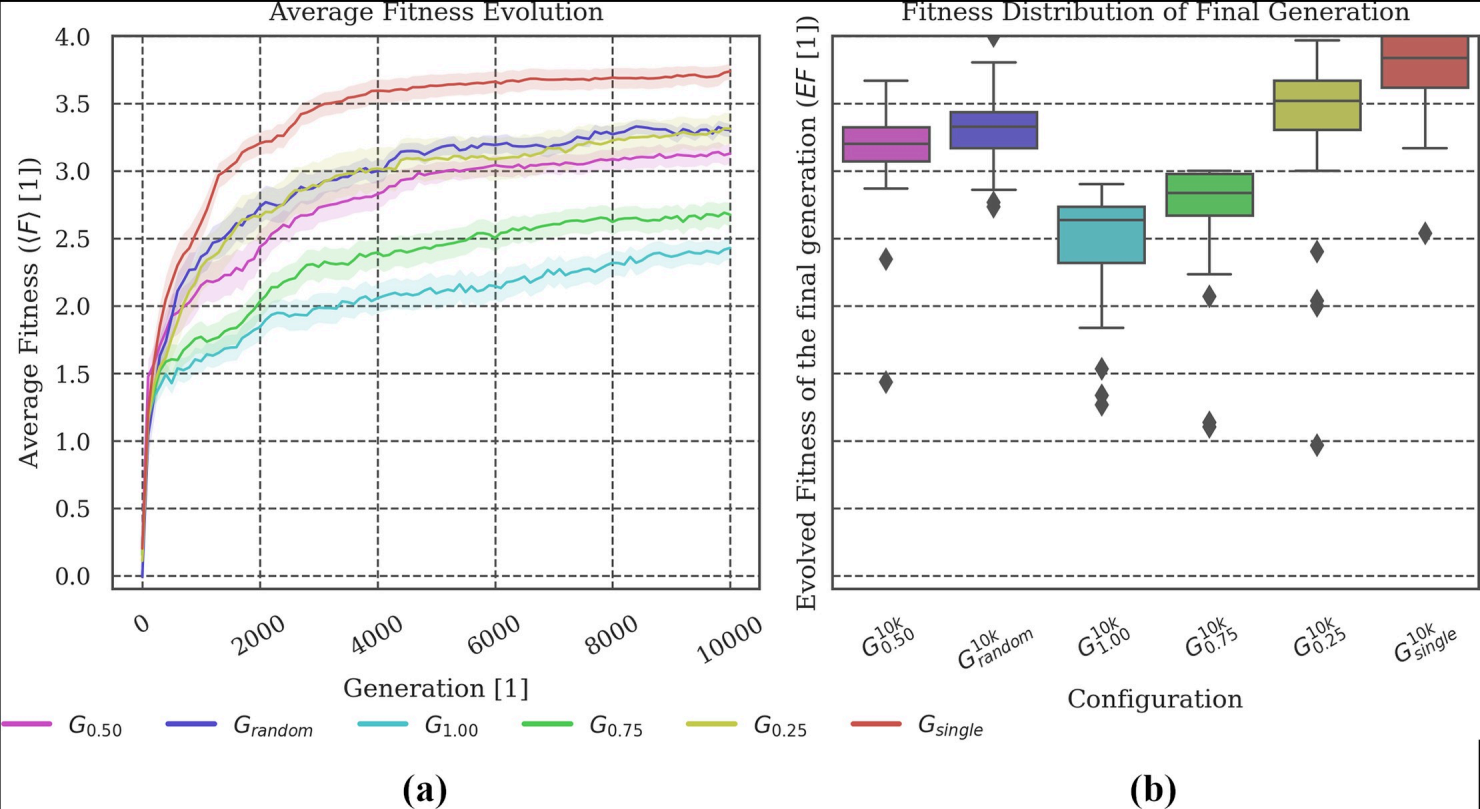
Fitness evolution and distribution of the final evolved fitness. **(a) *G***_***single***_ is the condition which evolves the highest fitness on average. Larger group sizes during evolution apparently impede the animats’ fitness evolution and lead to lower final evolved fitness values. **(b)** The evolutionary setup with randomized group sizes at each generation (***G***_***random***_) demonstrates similar properties as those setups with fixed, intermediate group sizes (***G***_***0*.*25***_ ***and G***_***0*.*50***_***)***.

**Fig 3:**
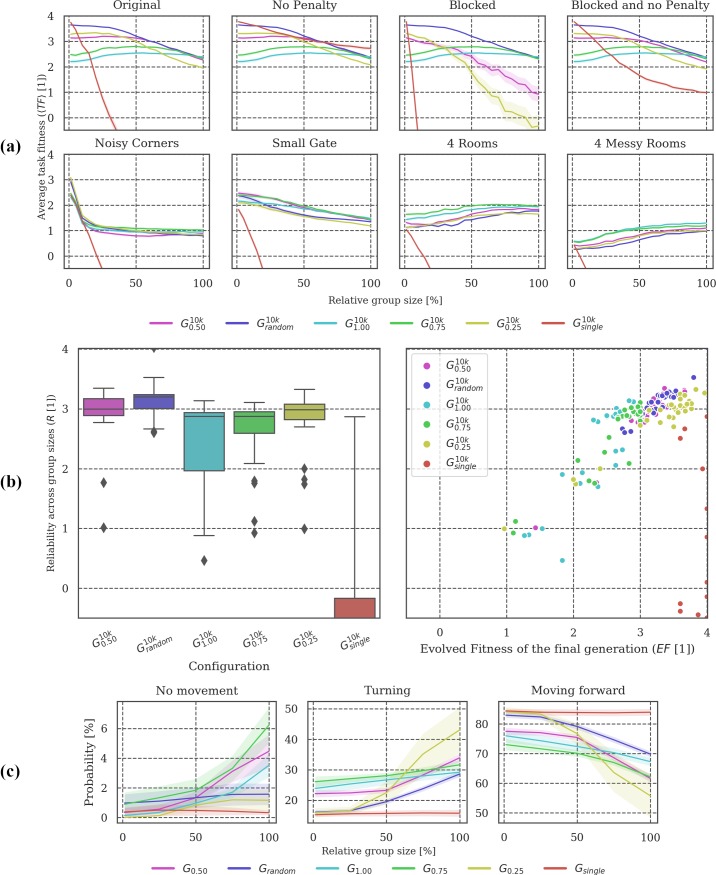
Post-evolutionary tests under modified conditions. **(a)** Overall, only ***G***_***single***_ failed to generalize across group sizes, presumably because animats that evolved without other group members did not develop strategies to avoid collisions (compare *Original* to *No penalty* test condition, where ***G***_***single***_ performs well throughout). There is a large difference in the *Blocked* environment between ***G***_***random***_, ***G***_***0*.*25***_, and ***G***_***0*.*50***_, while in other environments their task fitness is comparable, pointing to somewhat different navigation strategies. **(b)** On average, ***G***_***random***_ is the most reliable condition across varying group sizes, followed by ***G***_***0*.*50***_ and ***G***_***0*.*25***_. Except for ***G***_***single***_, ***EF*** correlates with ***R*** in all groups. **(c)** Note that ***G***_***0*.*50***_ and ***G***_***0*.*25***_ change their behavior more with increasing animat density compared to ***G***_***random***_.

**Fig 4:**
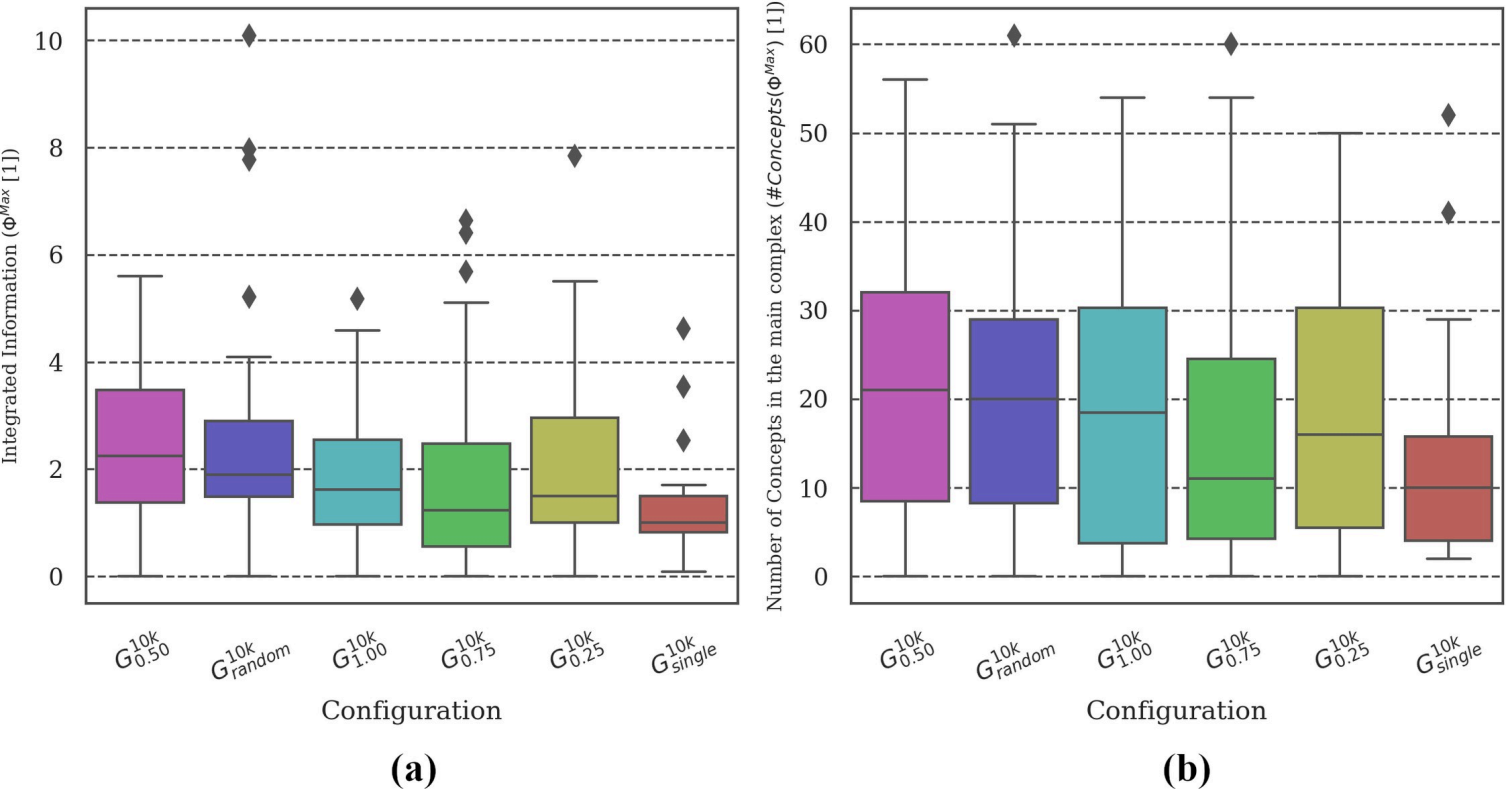
Distribution of brain complexity measures. Differences in **(a) *Φ***^***Max***^ and **(b)** the corresponding number of concepts was found between the most (***G***_***random***_ and ***G***_***0*.*50***_***)*** and the least (***G***_***single***_) reliable setups. Due to the large variance in the data and the low sample size (30 simulations per evolutionary setup), differences in the mean between the remaining conditions did not reach statistical significance (see Tables C and D in S1 Text).

**Fig 5:**
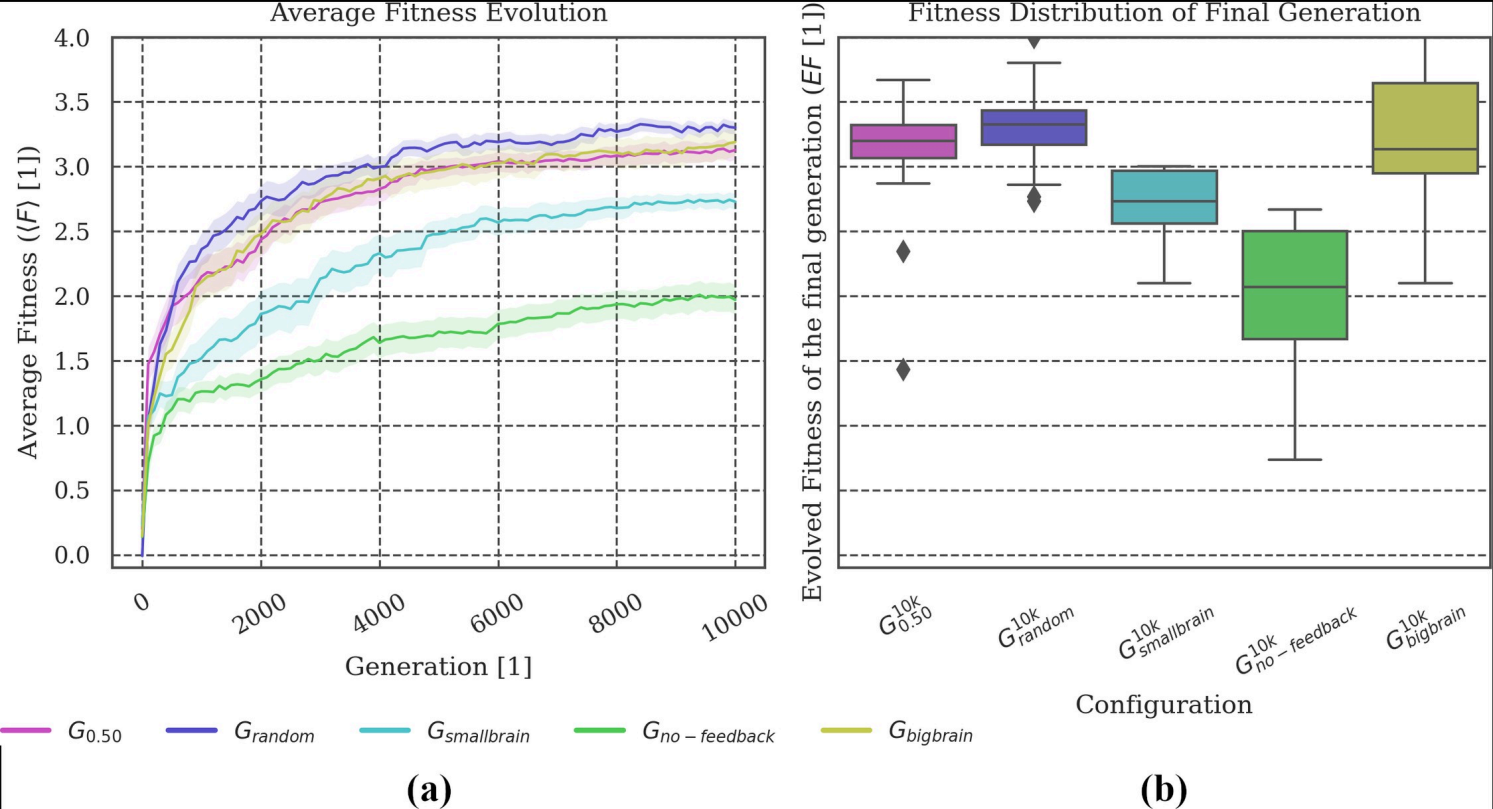
Fitness evolution and distribution of the final evolved fitness. **(a)** Less capacity for memory and internal computations impairs fitness evolution. Despite their similar capacity for memory, ***G***_***smallbrain***_ evolved higher task fitness than ***G***_***no-feedback***_. **(b)** Ceiling outliers suggest that animats in ***G***_***no-feedback***_ are generally capable of performing as well as the average animat in ***G***_***smallbrain***_ but that this is less likely. The performance of ***G***_***bigbrain***_ is comparable to ***G***_***0*.*50***_ with more distributed outcomes.

**Fig 6:**
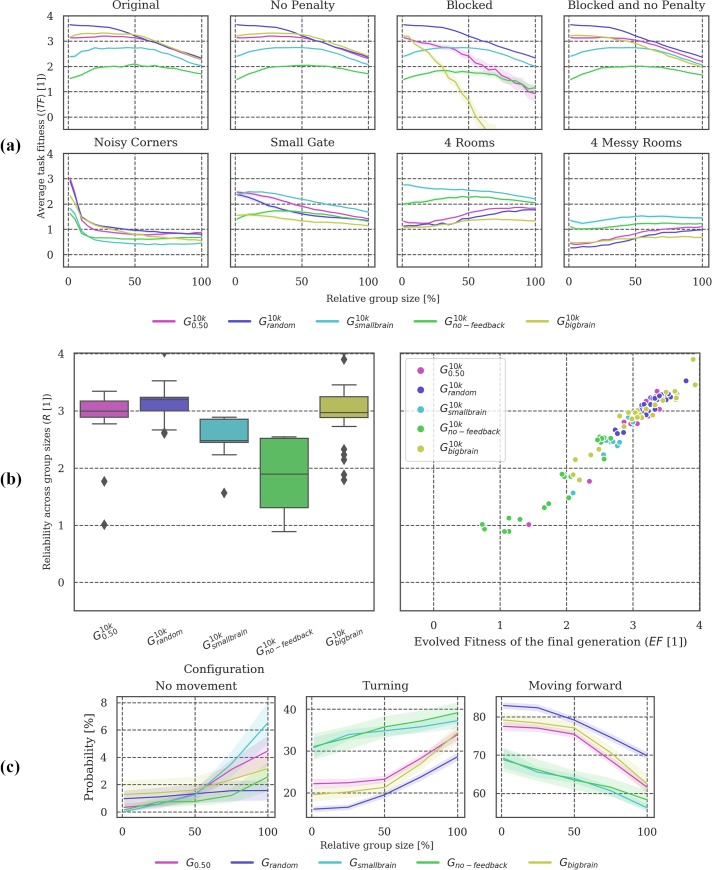
Post-evolutionary tests under modified conditions. **(a) *G***_***smallbrain***_ shows higher ***<TF>*** than ***G***_***no-feedback***_ across group sizes. ***G***_***bigbrain***_ is overall comparable to the baseline condition ***G***_***0*.*50***_, but shows worse performance in the *Blocked* test condition and some of the modified environments for larger group sizes. **(b)** Reliability ***R*** correlates with ***EF*** for all setups. The lower ***R*** values of ***G***_***smallbrain***_ and ***G***_***no-feedback***_ compared to baseline can thus be explained by their already lower evolved fitness values. Note, however, that ***G***_***smallbrain***_ and ***G***_***no-feedback***_ perform better than ***G***_***0*.*50***_ across group sizes in the *4 (Messy) Rooms* test conditions (see **(a)**). **(c)** For larger group sizes, ***G***_***smallbrain***_ remains static more often than ***G***_***no-feedback***_.

**Fig 7:**
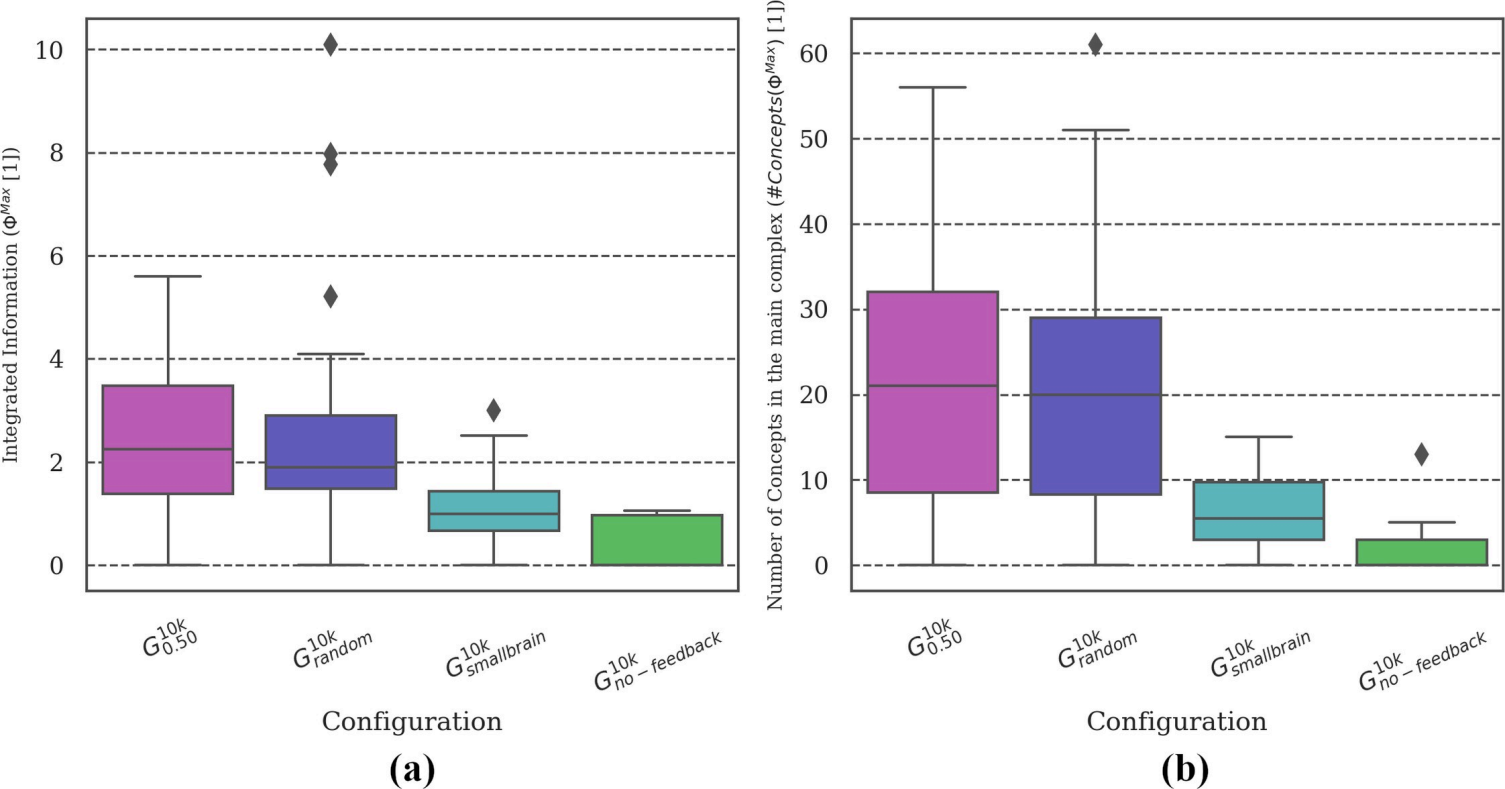
Distribution of brain complexity measures. Compared to the baseline, the smaller MBs (***G***_***smallbrain***_ and ***G***_***no-feedback***_) have lower ***Φ***^***Max***^ and fewer corresponding concepts. Animats in ***G***_***smallbrain***_ show higher ***Φ***^***Max***^ and have more corresponding concepts compared to ***G***_***no-feedback***_ animats, many of which have ***Φ***^***Max***^ ***= 0***. Due to computational reasons, the brain complexity of ***G***_***bigbrain***_ could not be calculated (see text).

**Fig 8:**
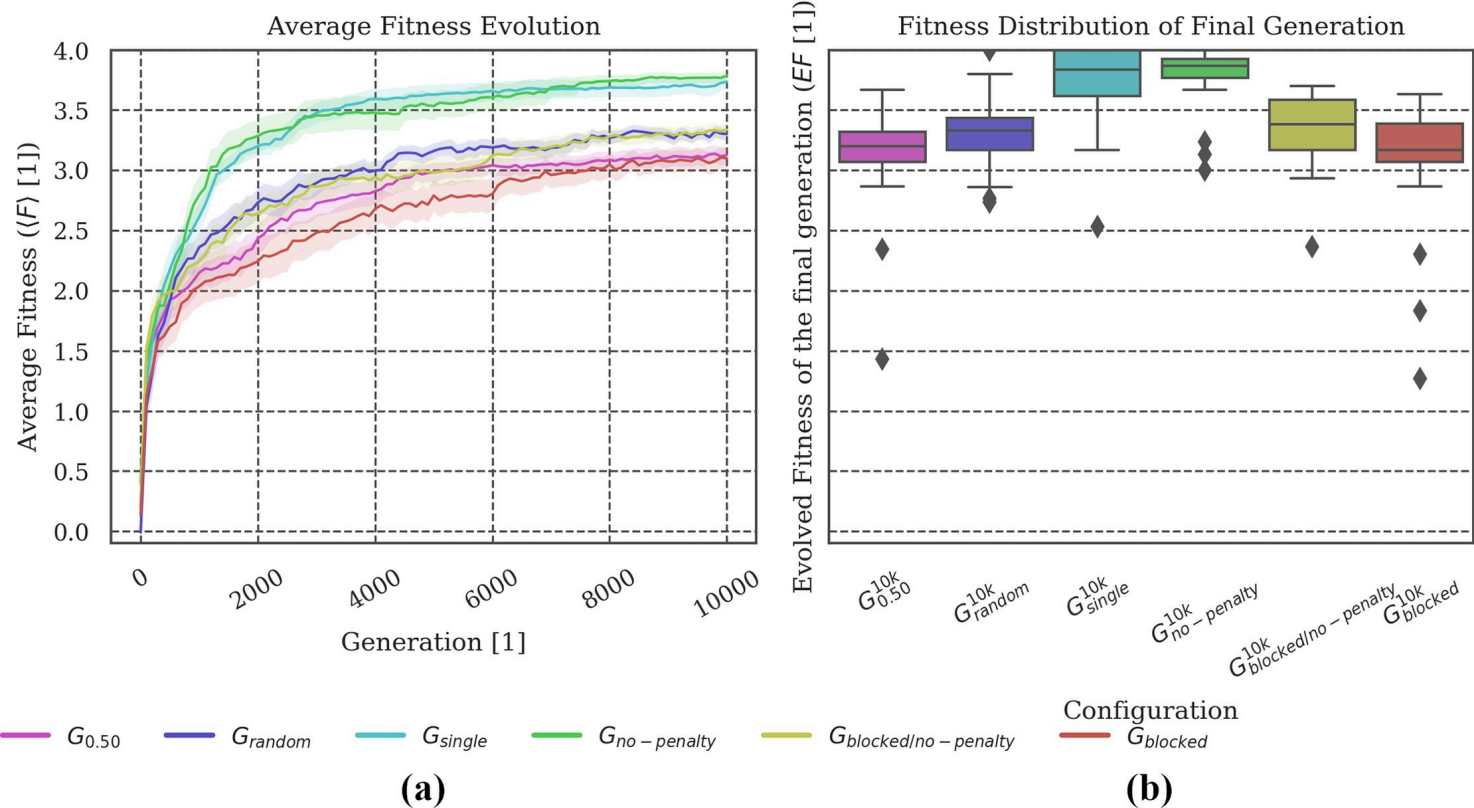
Fitness Evolution and distribution of the final evolved fitness. The animats in conditions without a penalty (***G***_***blocked/no-penalty***_ and ***G***_***no-penalty***_) evolved to relatively high fitness levels. In particular, ***G***_***no-penalty***_ evolved like ***G***_***single***_, which can be explained by the fact that animats in both of these conditions were not impacted at all by other animats. Similarly, ***G***_***blocked***_ seemed equivalent to the baseline setup ***G***_***0*.*50***_, while ***G***_***blocked/no-penalty***_ evolved to slightly higher fitness values, comparable to ***G***_***random***_.

**Fig 9:**
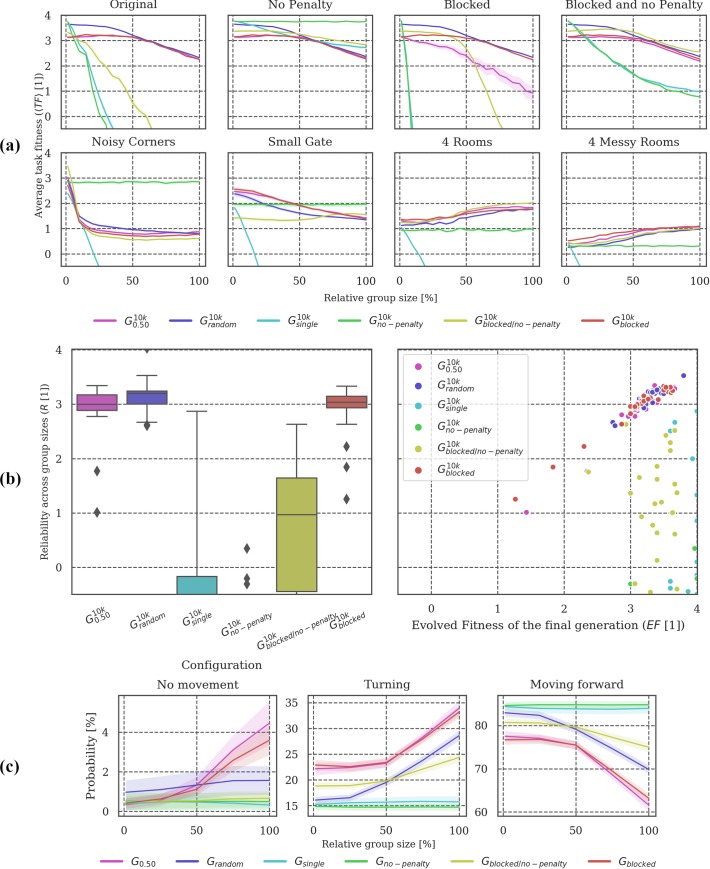
Post-evolutionary tests under modified conditions. **(a)** There was a significant difference between conditions in which interactions with other agents played a role for fitness evolution (***G***_***0*.*50***_, ***G***_***random***_, ***G***_***blocked*,**_ ***G***_***blocked/no-penalty***_) and those conditions in which it did not (***G***_***single***_ and ***G***_***no-penalty***_) (see text). **(b)** With a collision penalty imposed, ***G***_***no-penalty***_ showed similarly low reliability as ***G***_***single***_, whereas ***G***_***blocked***_ showed similarly high reliability as ***G***_***0*.*50***_. ***G***_***blocked/no-penalty***_ retained some reliability under collision penalty even though animats were evolved without it. **(c)** Similarities between ***G***_***0*.*50***_ and ***G***_***blocked***_, as well as ***G***_***single***_ and ***G***_***no-penalty***_ were also reflected in the animats’ behavior. The behavior of animats in ***G***_***blocked/no-penalty***_ was more reactive to changing group size than ***G***_***no-penalty***_.

**Fig 10:**
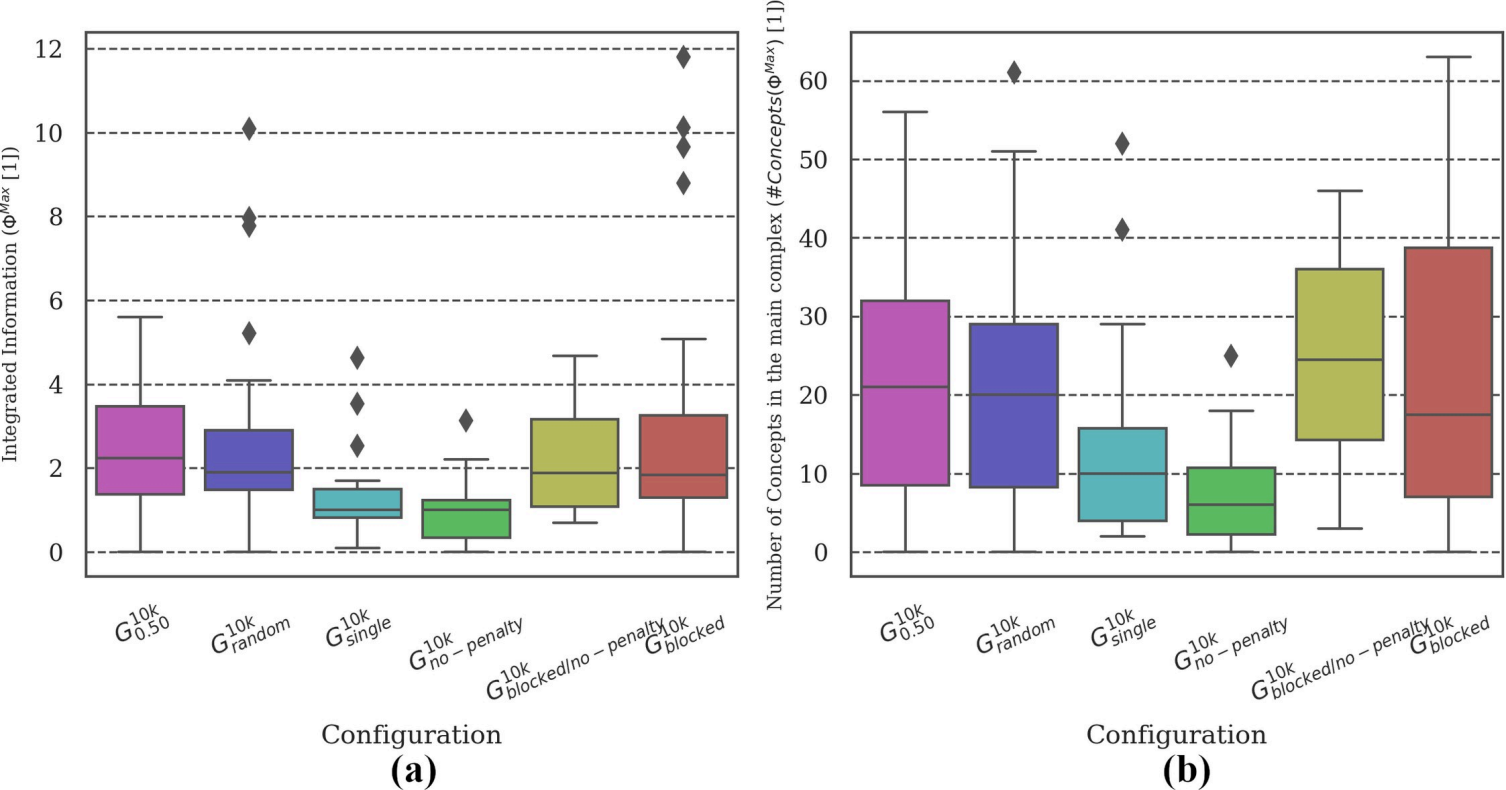
Distribution of brain complexity measures. In evolutionary setups where crossing each other was not possible (***G***_***blocked***_ and ***G***_***blocked/no-penalty***_), the brain complexity was comparable to the complexity of ***G***_***0*.*50***_. By contrast, animats in setups where the reaction to fellow animats had no reasonable effect on their performance (***G***_***single***_ and ***G***_***no-penalty***_) showed lower brain complexity. Still, there was high variance in the data of brain complexity.

**Fig 11:**
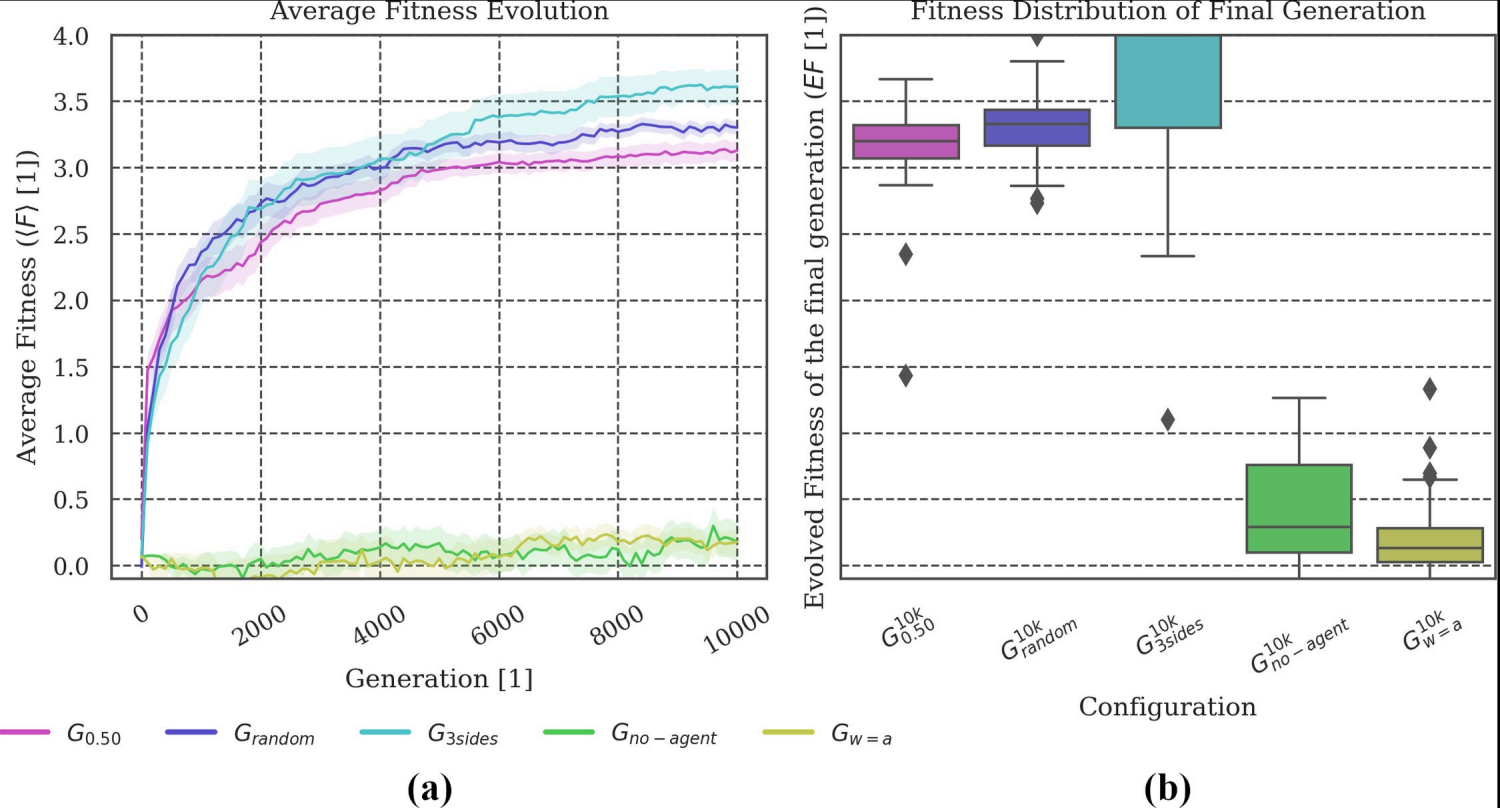
Fitness Evolution and distribution of the final evolved fitness. The average evolved fitness showed that animats in evolutionary setups without specific sensors for other animats (***G***_***no-agent***_ and ***G***_***w = a***_) achieved no reasonable fitness. By contrast, animats in ***G***_***3sides***_ outperformed ***G***_***0*.*50***_, and ***G***_***random***_, but also had more outliers with lower fitness and performed worse than the baseline condition ***G***_***0*.*50***_ in early generations (up to ~10,000 generations).

**Fig 12:**
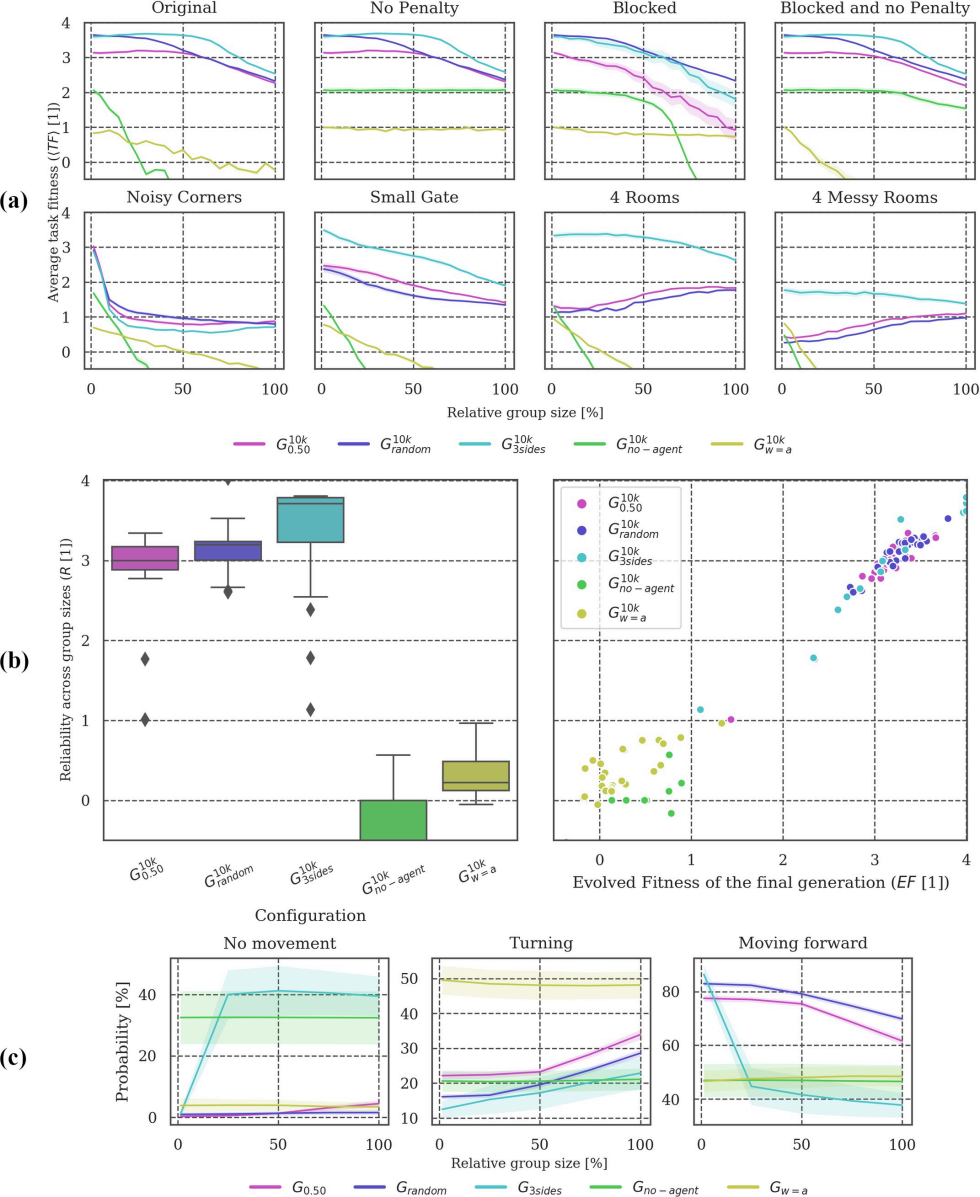
Post-evolutionary tests under modified conditions. **(a-b)** The ***G***_***3sides***_ condition had the highest 〈***TF***〉 in most test conditions, except in *Blocked* and *Noisy Corners*. In terms of ***R***, sensing everything (***G***_***w = a***_) with one sensor is still better than only sensing the walls (***G***_***no-agent***_). **(c)** Setups with few sensors evolved no typical behavior (high variance of movement between the 30 different evolutions, shaded area). The ***G***_***3sides***_ setup becomes more reactive as soon as the animat density starts to rise and thus evolved a different behavioral strategy than ***G***_***0*.*50***_ and ***G***_***random***_.

**Fig 13:**
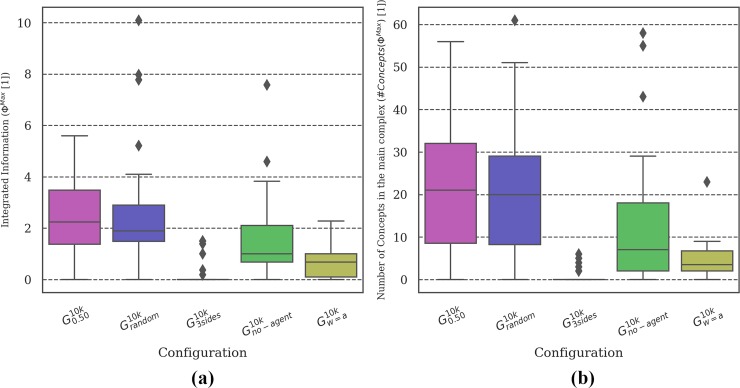
Distribution of brain complexity measures. Animats in the ***G***_***3sides***_ condition showed the lowest brain complexity of all setups despite having the highest evolved fitness and reliability. By contrast, animats with limited sensor information (***G***_***no-agent***_ and ***G***_***w = a***_) had lower than baseline complexity values, but also low evolved fitness (***EF***, see Fig 11).

We organized the presentation of our results into four sections categorized according to the evolutionary setups, as shown in Table 1 (varying “group size” (Figs 2–4), “cognitive architecture” (Figs 5–7), “interaction conditions” (Figs 8–10), and “sensor configuration” (Figs 11–13), respectively). Each section contains three figures displaying (1) the fitness evolution across generations and final evolved fitness values, (2) the task fitness, reliability, and behavioral features under modified post-evolutionary test condition (see Table 2), and (3) a complexity analysis of the evolved MBs. Since the figures are redundant in their construction, we will briefly introduce their attributes:

**Evolved fitness:** Figs 2, 5, 8 and 11 show (a) the mean fitness 〈***F***〉 evolution across generations and (b) the distribution of evolved fitness values (*EF*) of the final generation across the ***N*** = **30** evolution simulations that we performed per evolutionary setup. The shaded areas in (a) visualize the *standard error of the mean (SEM)*. The boxplots in (b) visualize the evolved fitness per condition ***G***_***i***_:

$$EF=F(A_{10,000}^{i}),$$ (1)

Where $A_{10,000}^{i}$ is the group of animats of the final generation of evolution simulation ***i***∈***N*** and $F(A_{10,000}^{i})$ its fitness value (see Methods for more details on the fitness function).

**Post-evolutionary tests:** Figs 3, 6, 9 and 12 visualize the results of testing the final generation of animats across different group sizes (***GS = [1*, *4*, *7*, *…*, *65*, *68*, *72]****)*, Panel (a) in Figs 3, 6, 9 and 12, shows the mean task fitness ***〈TF〉*** of testing the animats under different group sizes in their original environment and under additional modifications of the interaction conditions between animats or the environment design, listed in Table 2. Note that the condition under which a group of animats evolved is indicated by their ***G***_***i***_ label (see Table 1). ***〈TF〉*** is an average fitness across the ***N*** = **30** evolution simulations per experimental setup for a specific group size ***GS*** and (modified) condition ***M***:

$$\langle {\left(TF\right)}_{GS}^{M}\rangle =\frac{\sum_{i=1}^{N}F_{GS}^{M}(A_{10,000}^{i})}{N}.$$ (2)

Next, we quantified reliability for one test dimension, across modified group sizes in the “*Original*” test condition. We denote this specific measure of reliability as ***R***, computed as:

$$R={\langle {\left(TF\right)}_{Original}\rangle }^{GS}=\frac{\sum_{g}F_{g}(A_{10,000}^{i})}{\left|GS\right|}.$$ (3)

Note that in this case, the average is calculated across group sizes not evolution simulations as indicated by the subscript “***GS***”, which stands for group size with ***|GS| = 21*** (see above). Panel (b) shows the distribution of these reliability values (***R***) and their dependency on evolved fitness (***EF***). Finally, panel (c) shows how the animats’ behavior depends on the relative group size in the “Original” test environment, evaluating the probability of an animat to stand still (“no movement”), turn, or move forward. Percentages are displayed in a scale from 0–100%.

**MB Complexity analysis:** Figs 4, 7, 10 and 13 show two types of metrics for MB complexity: (a) the distribution of *integrated information* (***Φ***^***Max***^) [19,20], and (b) the corresponding *number of concepts* (***#Concepts(Φ***^***Max***^***)***) [19] per evolutionary setup. ***Φ*** and ***#Concepts(Φ***^***Max***^***)*** are dimensionless quantities and therefore have no unit.

### Varying group size: Evolution under specialized conditions can produce reliable agents

In a first set of experiments, we compared animats that evolved within groups of different, fixed sizes (***1–72*** animats), using the baseline animat and environment design in all cases, see Table 1: ***G***_***1*.*0-single***_. Preliminary results, including a comparison of the reliability ***R*** of evolution conditions ***G***_***1*.*0-single***_, were presented in [18]. As shown in Fig 2(A) and reported in [18], group size during evolution does impact the animats’ ability to perform the gate crossing task (see Fig 1(A)), which impacts the final evolved fitness ***EF***.

In our spatial-navigation task, animats in condition ***G***_***single***_ (group size of 1 animat) frequently find an optimal solution within 10,000 generations. We assume that this is due to the decreased difficulty of the task in this condition since colliding is impossible, and walls (static obstacles) may still guide the animat towards the gate. Increasing the number of animats in the environment seems to make it more difficult to navigate. Animats have to develop not only the ability to cross the gate, but also to avoid collisions with other group members, which would cause a penalty [18]. Reliability ***R*** across group sizes was found to be high if the animats evolved in an environment where the density of animats was balanced (***G***_***0*.*50***_ and ***G***_***0*.*25***_) (see (Fig 3A and 3B) and [18]).

In our study, we included an additional comparison setup (***G***_***random***_), for which group size varied randomly during evolution. We hypothesized that animats evolved in this setup should achieve high reliability ***R*** in the post-evolutionary tests since variation in group size would already be part of their evolution. As shown in Fig 2(B), the final fitness values ***EF*** for ***G***_***random***_ were comparable to those evolution setups with fixed, intermediate group sizes (***G***_***0*.*50***_ and ***G***_***0*.*25***_)–though still significantly different (***p <* .*05)***, see Tables A-G in S1 Text) for all statistical tests).

As hypothesized, ***R*** was found to be highest for ***G***_***random***_ (see Fig 3). Notably, however, animats that evolved under specialized conditions with intermediate group sizes (***G***_***0*.*50***_ and ***G***_***0*.*25***_) reached ***R*** values comparable to animats that already encountered variable group sizes during evolution (***G***_***random***_) (see Fig 3). ***G***_***0*.*50***_ and ***G***_***random***_ show similar 〈***TF***〉 values in the original environment setting, particularly for larger group sizes (***> 50%*** relative group size) (see Fig 3(A)). Nevertheless, ***G***_***random***_ animats evolved to higher ***TF*** for smaller group sizes, leading to comparable but still significantly different average ***R*** values (***p <* .*05***) (see Fig 3(B)).

While ***R*** quantifies reliability across modified group sizes in the *Original* test condition, the other post-evolutionary tests (see Table 2) may reveal further differences between evolutionary setups. For example, *Blocked* (in which animats cannot overlap) suggests a difference in strategy between ***G***_***0*.*50***_, ***G***_***0*.*25***_, and ***G***_***random***_ (see Fig 3(A)): ***G***_***0*.*50***_ and ***G***_***0*.*25***_ are more severely affected by this deviation from baseline settings in which animats can overlap, albeit under a penalty. While animats evolved in ***G***_***random***_ also experienced large group sizes with a higher likelihood of a penalty during evolution, ***G***_***0*.*50***_ and ***G***_***0*.*25***_ animats consistently faced only intermediate probabilities of colliding with other animats, which may have led to less effective strategies for avoiding collisions. In addition to varying group sizes, we also tested the final generation of animats in four environments with different wall arrangements (see Fig 3(A), bottom row). 〈***TF***〉 decreased to similarly low levels in all conditions, but least for evolutionary setups with larger group sizes. Note also that ***G***_***random***_ demonstrated relatively low 〈***TF***〉 under modified wall arrangements. Thus, high reliability across one dimension (here, modified group sizes as evaluated by ***R***) does not necessarily transfer to other dimensions (e.g., modified wall arrangements).

In terms of their behavior (see Fig 3(C)), animats in ***G***_***random***_ were less idle and showed fewer turns and more steps forward in comparison with animats in ***G***_***0*.*50***_, particularly for large group sizes. This suggests that the movement in ***G***_***random***_ is more fluid overall (see also Table 3). By contrast, the specialized animats display larger differences in behavior across group sizes. Please refer to [18] for a more detailed discussion of behavioral differences across evolutionary setups with fixed group sizes ***G***_***1*.*0-single***_.

**Table 3:** Absolute difference between the state transition probability *P* of *G*_*0*.*50*_ and *G*_*random*_ (*P(G*_*0*.*50*_*)–P(G*_*random*_*)*). The first digit (***S***) describes whether anything (wall or other animat) is sensed (1) or not sensed (0), and the second digit (***M***) describes whether the animat moved/turned (1) or did not move/turn (0). Most notably, ***G***_***random***_ animats performed more movements even in the absence of sensor inputs than ***G***_***0*.*50***_ (“01→01”).

*SM*		t+1			
		00	01	10	11
**t**	**00**	0.0000	-0.0074	0.0000	-0.0001
	**01**	-0.0079	-0.0606^1^	0.0136	0.0088
	**10**	0.0005	0.0100	0.0063	0.0063
	**11**	-0.0001	0.0119	0.0031	0.0157

^1^ Negative values indicate that the transition is more frequent in ***G***_***random***_, while positive values indicate the opposite.

Fig 4 shows the distribution of ***Φ***^***Max***^ and ***#Concepts(Φ***^***Max***^***)*** [19,20] as a measure of the complexity of the evolved MBs across evolutionary setups with different group sizes ***G***_***single-1*.*0***_ and ***G***_***random***_. While the evolutionary setups with the highest ***R*** values (***G***_***random***_ and ***G***_***0*.*50***_) do show the highest average values of ***Φ***^***Max***^ and the largest number of concepts (internal mechanisms), differences between conditions generally do not reach statistical significance (***p> =* .*05***) due to the large variance in the complexity values (see Tables C and D in S1 Text). We assume that it would require more data (simulation experiments per evolutionary setup) to refine the mean of the intervals enough to verify the observed trend. In our predecessor study [18], a correlation of high evolved fitness ***EF*** and reliability ***R*** with high brain complexity was found using a simplified measure of brain complexity based on anatomical connectivity only. The integrated information measures employed here are sensitive to the causal interactions within the MBs and thus also capture functional aspects in addition [19,20] In the present data, significant pair-wise differences could be found between ***G***_***single***_ and the most reliable setups (***G***_***random***_ and ***G***_***0*.*50***_). As explained above, the task environment experienced by animats in ***G***_***single***_ is less demanding than for setups with larger group sizes. Our observations are thus in line with [20], which demonstrated higher ***Φ***^***Max***^ and ***#Concepts(Φ***^***Max***^***)*** for animats evolved in more complex environments.

### Varying cognitive architecture: Brain size and memory dependencies

In a second set of experiments, we used the same environmental setup as for ***G***_***0*.*50***_ in all tested conditions, but varied the number of available computational units in the animats’ MBs. In the baseline design ***G***_***0*.*50***_, it is possible for the motor units to act as additional memory units (see Methods section). In one condition, ***G***_***no-feedback***_, the ability of the motor units to provide feedback was disabled, which reduced the absolute capacity for memory from six to four binary units. Moreover, we designed animats with similarly small memory capacity but with feedback motors as a reference group (***G***_***smallbrain***_). Those animats had the original type of motors with the possibility of evolving feedback loops, but only two memory units instead of four. Finally, we included a condition with larger MBs with eight memory units and motor feedback (***G***_***bigbrain***_).

We observed that evolved fitness ***EF*** and reliability ***R*** across group sizes in the original environment decreased for animats with fewer memory units (see Figs 5 and 6). However, while animats in ***G***_***smallbrain***_ still evolved to reasonably high fitness and reliability, ***G***_***no-feedback***_ was lacking in both. This observation indicates that motor feedback facilitates evolution in our task environment. One reason could be the fact that motor feedback allows the animats to utilize information about past movements directly (*e*.*g*., *like the sensation of one’s legs*). One behavioral difference between ***G***_***no-feedback***_ and ***G***_***smallbrain***_ was the reduced movement in the animats of ***G***_***smallbrain***_ (see Fig 6(C)). Furthermore, the state transition analysis shows that the motor units of animats in ***G***_***smallbrain***_ tend to change their behavior more often, while animats in ***G***_***no-feedback***_ stay in the same state more often (see Table 4). Notably, ***G***_***no-feedback***_ and, particularly, ***G***_***smallbrain***_ performed better than ***G***_***0*.*50***_ in the *4 Rooms* and *4 Messy Rooms* test conditions (see Fig 6(A), bottom row).

**Table 4:** Absolute difference between the state transition probability *P* of *G*_*smallbrain*_ and *G*_*no-feedback*_ (*P(G*_*smallbrain*_*)–P(G*_*no-feedback*_*)*). The first digit (***S***) describes whether anything (wall or other animat) is sensed (1) or not sensed (0) and the second digit (***M***) describes whether the animat moved/turned (1) or did not move/turn (0). Most notably, animats in ***G***_***smallbrain***_ switched more often between sensing and moving than animats in ***G***_***nofeedback***_ (“01→10”, “10→01”, but “11→11”).

*SM*		t+1			
		00	01	10	11
**t**	**00**	0.0000	0.0001	0.0000	0.0000
	**01**	0.0000	-0.0167^1^	0.0237	-0.0046
	**10**	0.0000	0.0194	0.0011	0.0029
	**11**	0.0001	-0.0004	-0.0015	-0.0241

^1^ Negative values indicate that the transition is more frequent in ***G***_***no-feedback***_, while positive values indicate the opposite.

By contrast, more memory units (***G***_***bigbrain***_) do not improve the fitness evolution or the task fitness ***TF*** in any of the tested conditions (see Figs 5 and 6). While ***G***_***bigbrain***_ achieves similar results compared to the baseline setup ***G***_***0*.*50***_, differences can be observed in the *Blocked* and *Small Gate* test conditions, as well as *4 (Messy) Rooms* for large group sizes (see Fig 6(A)). In principle more computational units should allow for better performance. However, the larger space of possible solutions may also impede fitness evolution (note the larger variance for ***G***_***bigbrain***_ compared to ***G***_***0*.*50***_ in Fig 5(B) and Fig 6(B)). Here, this trade-off may explain the similar mean 〈***EF***〉 and ***R*** values for ***G***_***0*.*50***_ and ***G***_***bigbrain***_.

Considering brain complexity, the evolutionary setups with smaller MBs (***G***_***smallbrain***_ and ***G***_***no-feedback***_) have significantly lower ***Φ***^***Max***^ and fewer concepts than the baseline condition (***G***_***0*.*50***_). Between those two conditions, ***G***_***smallbrain***_ shows significantly higher ***Φ***^***Max***^ and more concepts as compared to ***G***_***no-feedback***_ (see Fig 7). This correlates with the larger evolved fitness values of ***G***_***smallbrain***_ in Fig 5 and its associated higher reliability ***R*** in Fig 6. Note that calculating ***Φ***^***Max***^ and the corresponding number of concepts was not possible for ***G***_***bigbrain***_ since exhaustive evaluations across many systems and states are not currently feasible when using the *pyphi* software package to compute measures of integrated information theory for networks of that size (***>10*** units) [22].

### Varying interaction conditions: Evolution of beneficial interaction

In our baseline configuration for the evolution simulations (***G***_***0*.*50***_), individuals could occupy the same physical location but received penalties for colliding with other group members (see Methods section). We manipulated these features in the third set of simulations to evaluate how they influence both evolved fitness and reliability. Specifically, we considered three additional evolutionary setups: ***G***_***no-penalty***_, ***G***_***blocked***_, and ***G***_***blocked/no-penalty***_ (see Table 1 for a detailed description). ***G***_***single***_, ***G***_***random***_, and ***G***_***0*.*50***_ are also included in the figures for comparison.

Among the novel setups, only animats in ***G***_***blocked***_ were subject to the collision penalty during evolution. Not being able to share the same position (as in ***G***_***blocked***_) hardly influenced the evolved fitness ***EF***, the mean task fitness 〈***TF***〉 across post-evolutionary conditions, or the behavior of the evolved animats compared to ***G***_***0*.*50***_ (see Figs 8 and 9). Likewise, ***G***_***no-penalty***_, where reacting to other animats had no direct effect on the fitness evolution, showed very similar ***EF***, 〈***TF***〉, and behavior as ***G***_***single***_, with one exception: 〈***TF***〉 decreased with increasing group size in the *No Penalty* test condition for ***G***_***single***_ but not for ***G***_***no-penalty***_ which had evolved with a group size of 36 animats, as in ***G***_***0*.*50***_ (see Fig 9(A)). Note that ***R*** in Fig 9(B) was evaluated in the *Original* task condition with penalty, as for all other simulations sets.

Considering the post-evolutionary tests in Fig 9(A), the top row shows 〈***TF***〉 across group sizes in the *Original* environment (with penalty) and under varying interaction conditions: *No Penalty*, *Blocked*, and both *Blocked and no Penalty* (from left to right). In the bottom row of Fig 9(A), animats are evaluated under the same interaction rules as they evolved in while only facing a modified environment (position of static obstacles).

In this context, it is noticeable that ***G***_***no-penalty***_ performed relatively poorly for larger group sizes when tested in *4 (Messy) Rooms* despite receiving no penalty for collisions. By contrast, in evolutionary setups with a collision penalty and/or blocking 〈***TF***〉 increased with group size in the *4 (Messy) Rooms* test conditions. The decline in 〈***TF***〉 of ***G***_***blocked/no-penalty***_ for larger group sizes under test conditions with a collision penalty (*Original* and *Blocked*) moreover, suggests that these animats did not avoid physical interactions with their group members. However, even ***G***_***blocked/no-penalty***_ animats had an advantage compared to ***G***_***no-penalty***_ in the *4 (Messy) Rooms* environment. Taken together, these observations let us assume, that any evolutionary pressure to “pay attention” to fellow animats (through blocking or a collision penalty) could lead to the evolution of interaction strategies with possible advantages under certain (modified) conditions (e.g., using other animats for orientation or guidance).

Considering the brain complexity of animats in ***G***_***blocked***_ and ***G***_***blocked/no-penalty***_, we can report similar values compared to ***G***_***0*.*50***_ (see Fig 10). In summary, whether animats received a penalty for crossing each other, or whether crossing was prohibited to start with, did not significantly affect their evolved fitness, reliability, behavior, or brain complexity. Likewise, the brain complexity measures and behavioral results for ***G***_***no-penalty***_ were comparable to those of ***G***_***single***_.

### Varying sensor configuration: Sensory capacity influences reliability and brain complexity

We manipulated the animats’ sensor configuration (see Table 1) in a final set of evolution simulations. In addition to the baseline architecture (front wall sensor and front agent sensor), we designed animats with sensors on three sides ***G***_***3sides***_ (front, left and right wall and agent sensors), without an agent sensor ***G***_***no-agent***_ (one front wall sensor only) and with one universal sensor ***G***_***w = a***_ (sensing wall and agent as indiscriminate obstacles). Fig 11 reveals that our task environment required the ability to sense nearby animats and to differentiate between walls and animats in order to evolve reasonable ***EF*** values. Moreover, animats equipped with sensors on more sides achieved both higher evolved fitness ***EF*** and higher reliability ***R*** across group sizes than the baseline setup ***G***_***0*.*50***_ and ***G***_***random***_ (see Fig 11 and Fig 12B).

Overall, animats in the ***G***_***3sides***_ condition consistently outperformed the animats in other groups except in two test conditions: *Blocked* and *Noisy Corners* (see Fig 12A). This shows that animats which are equipped with more sensors do have an advantage on average, but they may still perform worse than animats with fewer sensors under special circumstances (here: *Noisy Corners*). We assume that the sensory signals in these specific environments might have been too different from the information patterns the animats evolved in and were thus specialized for. Nevertheless, the additional sensors led to high reliability ***R*** across group sizes as well as relatively high task fitness for most modified wall-arrangements even though the animats evolved under a specific group size and a fixed wall configuration (see Fig 12A and 12B).

While ***G***_***w = a***_ animats had only one sensor which does not discriminate between the wall and other animats, ***G***_***no-agent***_ was missing the animat sensor completely. Still, ***G***_***no-agent***_ showed better task fitness than ***G***_***w = a***_ in test conditions with small group sizes and without a penalty. Considering the evolved behavior, ***G***_***w = a***_ animats (see Fig 12(C)) were not reactive to other animats, which suggests that they did not evolve the capacity to differentiate between the animats and the walls internally, e.g., through memory. While ***G***_***w = a***_ and ***G***_***no-agent***_ moved forward at similar rates, ***G***_***w = a***_ performed proportionally more turns than ***G***_***no-agent***_, which stood still more often.

Analyzing the brain complexity showed that animats equipped with fewer, but also with more sensors than in the baseline setup ***G***_***0*.*50***_ evolved MBs with lower complexity (see Fig 13), albeit for different reasons. Based on the very low evolved fitness for ***G***_***w = a***_ and ***G***_***no-agent***_ (see Fig 11) we conclude that their MBs did not develop the necessary structure and mechanisms to solve the task, as reflected by their low brain complexity. By contrast, animats in ***G***_***3sides***_ achieved high ***EF***, ***<TF>***, and reliability ***R*** across group sizes, but did not evolve any integrated information (***Φ***^***Max***^ ***= 0***) in most cases. This observation was in line with previous findings on the relation between sensory capacity and internal complexity [20] and suggested that high brain complexity in cognitive systems depends on a need for internal memory and computation, which may decrease if an animat is equipped with more sensors.

## Discussion

The evolution of cooperative multi-agent systems might be the next frontier in the context of evolving artificial agents. To date, however, not much is known about conditions that give rise to cooperative behavior and the complex inter-dependencies between individual and group goals [26]. For example, there might be many factors that influence whether the individuals either bow to the group or act by egoistic rules [27]. In this study, we used animats equipped with MBs (introduced by Edlund et al. [24]) to study how group performance and its reliability under modified conditions depends on the individual, interactions between individuals, as well as specific features of the MBs’ evolution.

### Prior work investigating group evolution

Earlier research that implemented groups of MBs concentrated on predator-prey environments and showed that animats can (co-)evolve swarm behavior [28–30]. The animat design in this work was generally based on a design in Marstaller et al. [16], who evolved individual MBs with the goal of solving perceptual-categorization tasks. Another method of simulating swarm behavior is neuro-evolution, i.e., the evolution of *artificial neural networks (ANN)* [31–33]. As in Olson et al. [29], these neuro-evolution experiments produced agents which evolve in a swarm to solve a predator-prey task.

Other researchers have investigated the effect of group size in the evolution of groups of simulated agents beyond predator-prey scenarios in a more general context. They find that the behavior of the group of agents and the individual agent is dependent on the group size [34,35]. In another study which changed the group size during evolution, the authors show that it can be easier for smaller groups than larger ones to organize themselves [5].

The effect of changing swarm sizes has also been investigated in the context of natural biological systems: Brown [27] examined which factors are decisive for the individual to either join a swarm or behave egoistically. The study focused on experimenting with environmental qualities and swarm size. Brown defined *optimal swarm size* as the best trade-off between the advantage of balancing costs between individuals in the swarm and the disadvantage of sharing the resources (energy/food) with the whole swarm. In an earlier study, Pacala et al. [4] report that swarm size constrains information transfer and task allocation. They argue that the information exchange varies and the task allocation changes, depending on the swarm size of ant-colonies. Pacala et al. [4] also argue that swarm behavior is the product of social interaction, individual interaction, and the interaction with the given environment. In a more recent work [36], we found arguments that swarm behavior arises if there is sufficient density within the swarm.

### Factors that impact evolved fitness and reliability

Generally, the ability to evolve high fitness in a given evolutionary setup depends on the interplay between external and internal factors as, e.g., the complexity of the environment and the animats’ architecture (see also [20]). Exemplary for these factors, we manipulated the group size and the animats’ sensorimotor and memory capacities across evolutionary setups. Further, we evaluated how these manipulations affected fitness evolution and post-evolutionary reliability.

#### Different group sizes

In the specific evolutionary setup investigated here, evolved fitness ***EF*** negatively correlated with group size, which is a result of the imposed penalty for collisions with other group members (see Figs 2 and 8, animats that evolved without the risk of penalty (***G***_***single***_ and ***G***_***no-penalty***_) achieved the highest 〈***EF***〉). On the other hand, animats evolved in fixed, intermediate group sizes (e.g., ***G***_***0*.*50***_ and ***G***_***0*.*25***_) are most reliable to changes in group size as measured by ***R***, and, in fact, comparable to ***G***_***random***_, in which animats experienced random group sizes during evolution (see Fig 3(B)). The optimal group size for high ***R*** in our experiments is thus larger than the optimal group size for high ***EF***, or individual fitness. This observation suggests, more generally, that unexpected changes in group size during evolution may sometimes lead to larger group sizes than expected based on what is best for an individual within the group.

#### Capacity for memory

Animats with less capacity for memory (***G***_***smallbrain***_ and ***G***_***no-feedback***_) evolved to lower ***EF*** values than the baseline condition ***G***_***0*.*50***_ (see Fig 5). Further, the low memory setups were less reliable under changes in group size (low ***R***). A higher memory capacity as in ***G***_***bigbrain***_ did not provide further advantages compared to ***G***_***0*.*50***_. Given the higher variance of ***G***_***bigbrain***_ in ***EF*** and ***R***, we suspect that the larger search space made it more difficult for the evolutionary algorithm to converge to an optimal solution.

#### Sensorimotor capacity

Finally, more sensors (***G***_***3sides***_) proved advantageous for both evolved fitness ***EF***, reliability ***R*** across group sizes, and task fitness ***TF*** under almost all modified test conditions, including most modified wall arrangements (Fig 12(B)). By contrast, training animats on multiple group sizes during evolution (***G***_***random***_) led to high ***R***, but did not translate to high task performance under modified wall arrangements (Fig 3(B)). We speculate that the additional sensors allowed the animats to evolve more generalizable strategies in our two-dimensional spatial-navigation task, even though they evolved in a single static environment.

Note that we did not include a comparison condition in which animats evolved under various wall-arrangements, since it is not trivial to determine a statistically representative sample of all possible environments as part of the evolutionary simulation. For the same reason, we did not quantify average reliability across modified wall-arrangements, but provided task fitness measures for each tested wall-arrangement (Figs 3, 6, 9 and 12(A)). In addition, Table G in S1 Text lists 〈***TF***〉 values for all evolutionary setups and test environments evaluated in this study.

Overall, our findings suggest that, in general, animats that were well-equipped for dealing with their original task environment (and thus achieved high evolved fitness) also performed better under modified conditions that were never encountered during evolution. Within most evolutionary setups, reliability ***R*** was correlated with evolved fitness (see Figs 3, 6, 9 and 12(B), right panel). The only exceptions were ***G***_***single***_ and ***G***_***no-penalty***_, which did not adapt to the behavior of other group members at all. The high evolved fitness in ***G***_***single***_ and ***G***_***no-penalty***_ could thus be interpreted as a form of narrow intelligence. By comparison, intermediate group sizes led to a somewhat more general form of intelligence.

Nevertheless, our findings also show that evolutionary setups that seem less adapted (lower evolved fitness) overall may still have advantages under some special modifications. For example, animats evolved in larger groups (***G***_***1*.*00***_ and ***G***_***0*.*75***_) or with less memory capacity (***G***_***smallbrain***_ and ***G***_***no-feedback***_) performed better than ***G***_***0*.*50***_ under most modified wall-arrangements (see Figs 3 and 6(A), bottom row; Table G in S1 Text). On the other hand, even ***G***_***3sides***_ performed worse than the baseline (***G***_***0*.*50***_) in one of the modified test environments (*Noisy Corners*).

#### Interactions between individuals in the group

In this study, we did not explicitly implement any form of direct communication between animats. Nevertheless, we found that it was necessary for animats to perceive their fellow group members and to distinguish them from static obstacles to achieve reasonable evolved fitness ***EF*** and reliability ***R*** (see Figs 11 and 12, where both ***G***_***no-agent***_ and ***G***_***w = a***_ overall show low values). Moreover, we observed that evolved interaction strategies provided advantages under certain modified conditions: Animats that evolved without a collision penalty (***G***_***no-penalty***_) performed worse in some of the modified environments, even if tested without receiving a penalty (see Fig 9(A), *4 (Messy) Rooms*). While animats in ***G***_***no-penalty***_ were equipped with an agent sensor, they had no incentive to interact with or “pay attention” to their fellow agents. By contrast, the task fitness in the *4 (Messy) Rooms* conditions typically increased with group size for animats that evolved in groups and received either a collision penalty (e.g., ***G***_***0*.*25***_ –***G***_***1*.*0***_) and/or could not pass other agents (***G***_***blocked***_ and ***G***_***blocked/no-penalty***_) (see Figs 3(A) and 9(A)). This indicates that they may have used other agents for orientation or guidance, a form of implicit cooperation. Indeed, animats evolved in large groups (***G***_***0*.*75***_ and ***G***_***1*.*0***_) showed higher task fitness than ***G***_***0*.*50***_ in these particular modified test environments (see Fig 3(A), bottom; Table G in S1 Text).

As we know from previous studies, swarm behavior in nature can be the result of simple reactions to local neighbors [3,37]. For example, it could be a good strategy to stay close to a group member without hitting it. Such evolved behavior may then provide additional fitness advantages under some modified conditions (as in the *4 (Messy) Rooms* test condition here). The observed instances of cooperative behavior can thus be viewed as an emergent phenomenon of the evolutionary process.

### Relation between brain complexity, evolved fitness, and reliability

Previous studies applying measures of integrated information to adaptive animats equipped with MBs [20,24,38] have observed that, on average, ***Φ***^***Max***^ and related measures for brain complexity increase over the course of evolution, which correlates with increasing evolved fitness ***EF*** (see Table G in S1 Text). Moreover, as demonstrated in [20], this increase depends on the complexity of the environment relative to the animats’ sensor capacity: MBs that evolved in environments which require more memory and internal computation developed higher average ***Φ***^***Max***^ values and a higher number of concepts.

For the evolutionary setups with the baseline animat architecture as in ***G***_***0*.*50***_, we found the highest values of ***Φ***^***Max***^ and ***#Concepts(Φ***^***Max***^***)*** for medium group sizes ***G***_***0*.*50***_, ***G***_***blocked***_, and for ***G***_***random***_. These setups were also among the most reliable across group sizes (see also [18] for similar results using a simplified measure of brain complexity). By contrast, significantly lower ***Φ***^***Max***^ values were found for ***G***_***single***_ and ***G***_***no-penalty***_, the two setups in which task fitness during evolution did not depend on interactions with other animats. As argued above, ***G***_***single***_ and ***G***_***no-penalty***_ thus effectively evolved within a simpler task environment than ***G***_***0*.*50***_, ***G***_***blocked***_, and ***G***_***random***_, which explains their lower brain complexity ***Φ***^***Max***^.

Compared to ***G***_***0*.*50***_, evolutionary setups with altered animat architectures showed consistently lower values of ***Φ***^***Max***^ and ***#Concepts(Φ***^***Max***^***)***. Limiting the animats’ sensor capacity (***G***_***no-agent***_ and ***G***_***w = a***_) or the number of available memory units (***G***_***smallbrain***_ and ***G***_***no-feedback***_) interfered with their capacity for successful evolution in the spatial navigation task. Their lower evolved fitness was thus accompanied by less developed MBs with lower ***Φ***^***Max***^ and fewer concepts. Given more time to evolve (more generations), both their performance and their brain complexity might still increase. By contrast, more sensors allowed for better performance (***EF***, ***TF***, and ***R***) based on high amounts of external information, which effectively decreased the need for internal complexity (memory and computations) and thus may also lead to low ***Φ***^***Max***^, as observed here for ***G***_***3sides***_.

In theory, high fitness in any given environment could be achieved without information integration (***Φ***^***Max***^ = 0) if no restrictions are imposed on the animats’ architecture (e.g., by a system with a large feed-forward architecture [19]). Moreover, information integration can be high even if there is no reasonable fitness, which partially explains the large variance in the brain complexity measures (see, e.g., outliers for ***G***_***no-agent***_ in Fig 13). However, given a certain requirement for memory and context sensitivity, constraints in the number of sensors and memory elements may give rise to an empirical lower boundary on the amount of integrated information necessary to perform a given task [20,24,38,39].

In summary, for a given MB architecture, higher brain complexity seems to be related to better performance and reliability. However, future work should explore under which environmental conditions additional sensors, or more internal units, become more advantageous for the evolution of higher fitness (***EF***) and reliability (***R***).

## Limitations

Our work modeled one particular, small-scale scenario of a multi-agent evolutionary setting. Future work should consider other types of environments which may strengthen the generality of our results. Moreover, further evolution or training scenarios for artificial organisms should be considered as well—here we do not use crossover in the genetic algorithm, for example, and all animats placed in the same environment are clones. In addition, Markov Brains are just one type of computational substrate and it would be interesting to see whether other types of substrates (e.g. Artificial Neural Networks) behave differently under modified test conditions [40]. Nevertheless, the results obtained in our simulation study could also be directly compared against certain types of biological models (e.g. investigating the behavior of army ants under environmental modifications [36,37]).

While the measures that we employed to assess the complexity of the evolved MBs are theoretically motivated [19], they are also computationally very complex. This made it difficult to evaluate a larger sample size (number of evolution simulations) or to analyze the brain complexity of more generations (not only the final one). This is why alternative, approximate measures should be considered, too. For instance, the *largest strongly connected component* (and other graph metrics) can be used as a proxy for system integration and thus brain complexity [18]. Efficient approximations would also enable investigations into how brain complexity develops across generations as performed in [20] for slightly smaller MBs. Moreover, ***Φ***^***Max***^, and the associated number of concepts ***#Concepts(Φ***^***Max***^***)***, are causal measures that assess the degree to which the mechanisms within a MB are differentiated and integrated. Future work should also consider and explore alternative informational or dynamical measures (e.g., [41–43]). In this study, we concentrated on changes in task fitness and reliability under modified conditions, so the brain complexity analysis was not the subject of more in-depth investigation.

## Conclusion

It is challenging to remain reliable in a dynamic and volatile world while also trying to succeed in a given task. Investigating the characteristics of this reliability, especially with regards to cooperative behavior, might also be useful to develop implications and strategies for improving the reliability of individuals within larger organizations. Despite complex dependencies between the individual, the group, and the environment, our computational approach offers a way to investigate reliability in group behavior. Here, we were particularly interested in the question of how cognitive and environmental constraints influence the reliability of simulated animats in a group. We were able to isolate essential influencing factors to better understand possible positive and negative effects of changing group size, environment design, and individual cognitive ability on reliability and task fitness under modified conditions. In particular, our study suggests that balancing the number of individuals in a group may lead to higher reliability under unforeseen changes in group size, even if the task itself would be simpler with fewer group members.

Moreover, a minimal number of sensors, the ability and incentive to distinguish static obstacles from other group members, and a minimal number of memory units were required to achieve high evolved fitness and reliability in our specific evolution simulations. If these minimal requirements were met, reliability ***R*** across group sizes was found to correlate with evolved fitness across the tested evolutionary setups. Limited sensor information forced the animats to evolve more complex brain structures, especially for intermediate group sizes, which also demonstrated the most reliable behavior across group sizes. Nevertheless, the highest task fitness across most modified conditions (varying group sizes as well as modified wall-arrangements) was observed for the evolutionary setup with additional sensors, which did not require high internal complexity. Finally, we presented data that support the evolution of implicit cooperation between animats. In all, this research asserts that task efficiency and effectiveness is not the only goal in dynamic environments; task reliability is also worth striving for.

## Materials and methods

We used an evolutionary algorithm to generate simulated animats evolving in groups under various evolutionary setups (see Table 1), testing different animat architectures and evolutionary conditions to evolve animats having heterogeneous behavior, evolved fitness, and reliability. Afterwards, we conducted post-evolutionary tests to assess the reliability of the different evolutionary setups under modified conditions (see Table 2). This section explains the animat designs, the environment, the evolutionary simulations, and the experiment setup. We used *MABE (Modular Agent-Based Evolver)* [44] as a computational evolution framework with the same parameters as in previous work [18] (see Table in S1 Table).

We chose MBs as a simplified model of an artificial brain, since the basic idea of an MB is to emulate the recurrent connectivity structure found in real neural networks in a simple manner, while being complex enough to represent a cognitive system [16]. Furthermore, a recent study showed that MBs can be very compatible against variations of *artificial neural networks* and even showed higher performance in general [17]. Nevertheless, it would, in principle, also be possible to use a finite state machine [21], or artificial neural networks [32] to solve the kind of task investigated here.

Individual animats had to solve a two-dimensional spatial-navigation task in the presence of other animats (clones), thus forcing individuals to react to these other animats in order to reach a high fitness value. This task was a redesign by Fischer et al. [18] of a task environment initially developed by Koenig et al. [21]. An animat can usually differentiate between static (borders and walls) and dynamic objects (animats) in the environment through two distinct sensors. This design allowed for the evolution of social behavior based on passive interactions between animats (we observed, e.g., “waiting”, or “following” behavior).

### Animat architecture

**Fig 14:**
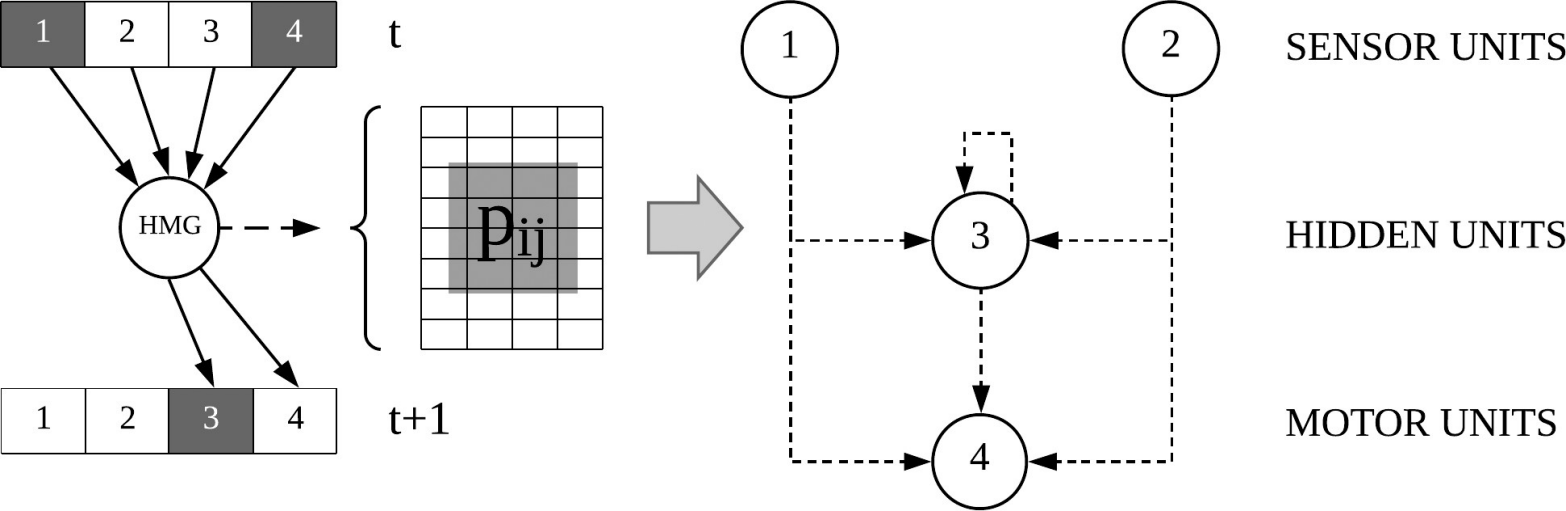
Example of an MB. An MB [24] has three components: (1) Units with a binary states (“1”-“4”), (2) HMGs and (3) the connections between the binary units and the HMGs. The connections between the units can be derived from the connections to the HMGs. HMGs contain the mechanism, e.g., a lookup table (here deterministic), to transform the brain state of units at ***t*** to the state at ***t+1***.

The evolutionary algorithm evolves animats with MBs, which contain a set of discrete, binary computational units (“neurons”). Each unit has its own update rules receiving inputs from and sending their output to other units. In this study, the decision system (the connectivity between units and their update-rules) was implemented by *Hidden Markov Gates (HMGs)*, which are encoded in an animat’s genome (string of integers [0–255] with a minimum length of 2,000 elements and a maximum length of 20,000 elements). The HMGs connect the nodes of the MB indirectly. Fig 14 visualizes a simple example, in which an HMG is connected to four units. The decision system inside an HMG can be diverse. In this research, we evolved discrete, deterministic lookup tables. The lookup tables translate the states of the connected input units at ***t*** to the new states of connected output units at ***t+1***. The motor or memory units can represent the output units of the HMG. The states of the sensor units are set by the input they receive from the environment.

The integers in an animat’s genome encode the HMGs: the number of HMGs, their lookup tables, the connected input units, and the connected output units. The MBs evolve by mutating the genome in each new generation (see [29,40]). Each locus in the genome mutated with a certain probability (point mutations). In addition, larger sections could be deleted or added to the genome [24,45] (again, all parameters are listed in Table in S1 Table). We did not use crossover or recombination (more than one parent per genome), since this would make it more difficult to trace an animat’s line of descent without obvious computational advantages in the simple evolutionary setting investigated here. In principle, other optimization algorithms could be employed to develop well-performing MBs. The evolutionary algorithm used here has the advantage that both the node connectivity and the nodes’ update rules can be encoded in the genome and jointly adapted through mutation and fitness selection.

**Fig 15:**
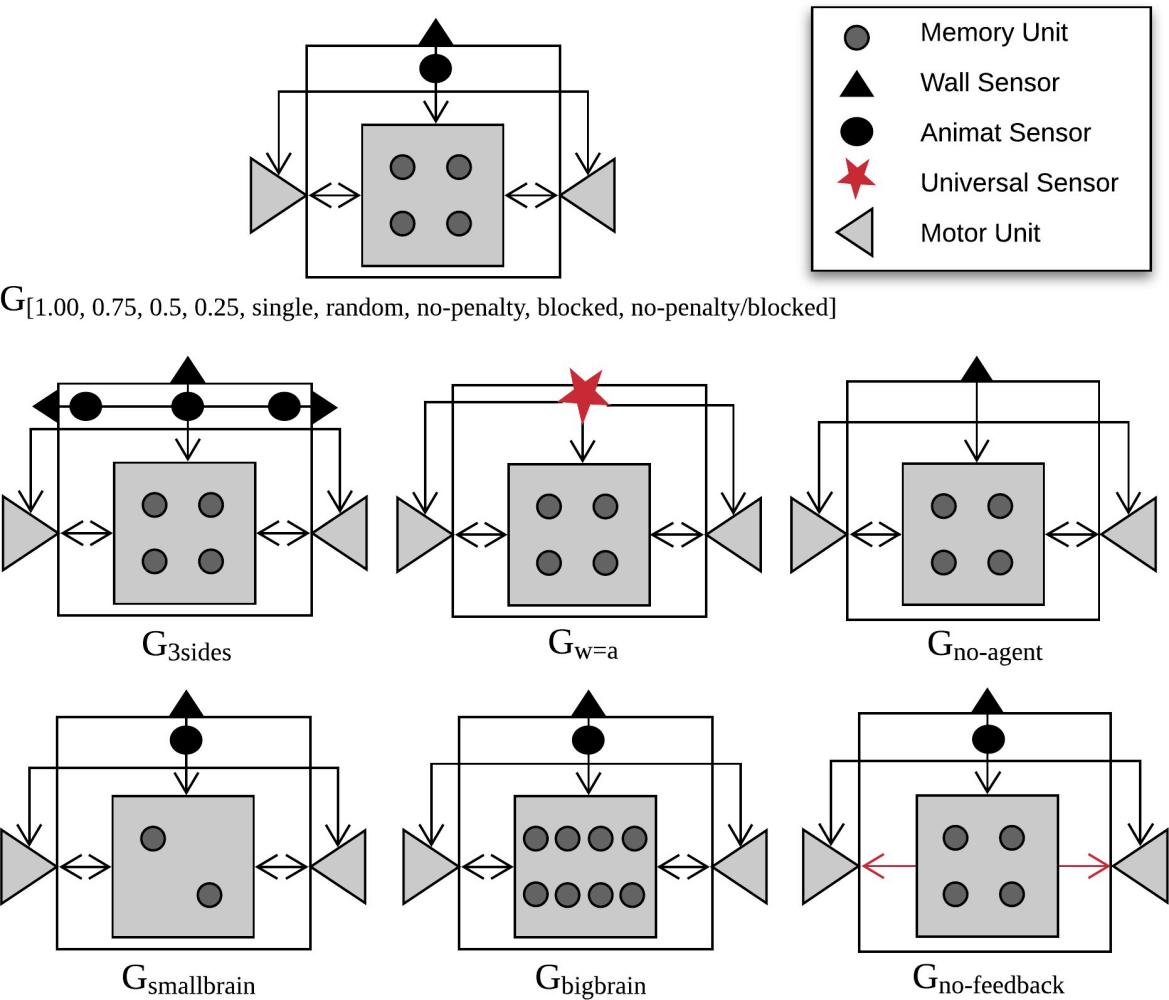
Schematic architecture of the five different animat architectures. The top row shows the *original* animat architecture as defined in [18]. The animats have two motor units (grey triangles), four memory units (dark grey circles) and one to six sensor units (black/red shapes). The middle row shows animats with a changed sensor architecture, from the left: The architecture with sensors on three sides, the architecture with a single sensor unit, detecting wall and animat indiscriminately, and the architecture without an animat sensor. The bottom row shows animats with changed memory architecture, from the left: The architecture with only two memory units, the architecture with eight memory units and the architecture without feedback motors (motors cannot be part of the memory network). Note that the architectures depict the maximal number of units available. Whether any given unit is actually used depends on the evolved connectivity and logic function. Animats are initialized in the first generation without connections between units.

All units in the animat’s MB have binary states, either ***1*** or ***0***. A sensor turns ***1*** if an obstacle is detected and a motor switches to ***1*** if it is active. Two motors provide the ability to turn 90 degrees left or right, and to move forward (if both motors are in state ***1***). Since the units within a MB can be interconnected in a recurrent manner, they have the potential to create internal memory. We evolved animats with five different animat designs displayed in Fig 15. The baseline cognitive architecture was introduced already in [18] (one front wall sensor, one front agent sensor, four memory units, and two motors). Here, further deviations were designed to investigate the influence of an animat’s sensorimotor and memory capacities on the resulting evolved fitness and the animats’ task fitness and reliability under modified post-evolutionary test conditions. The sensors had a detection range of one unit. Typically, the motor units could also feedback to the memory and motor units, thus acting as additional memory capacity, since knowledge about previous motor states is directly available for computing the next state. One animat design was included that lacked the possibility for motor feedback (***G***_***no-feedback***_).

### Design of the 2D environment

**Fig 16:**
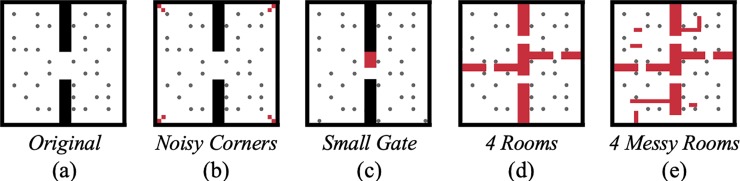
Environmental design. **(a)** The two-dimensional environment is based on a discrete grid architecture and contains two rooms. Animats draw a random starting position. Their orientation can be up, down, left, and right and is also randomly selected at initiation. **(b-e)** Four additional environments were used to test the task fitness of the animats under modified conditions. Red blocks mark the changes/additions in the room and represent walls. In (d), all four gates count as possible rewards. In (e), only gates on the vertical mid-line provide rewards.

All experiments simulated a two-dimensional environment. The world has ***32×32*** units (see Fig 16). All animats started on one of ***72*** predefined, uniformly distributed, starting positions. The selection for the starting position, as well as an animat’s initial orientation, was random at every new generation. The original environment (see Fig 16(A)) had two rooms, which are connected by a gate. The animats’ goal was to travel between the two rooms in order to achieve a high fitness value. This design was adapted from the work of Koenig et al. [21]. All evolutionary setups evolved in the original environment. As an additional test dimension for evaluating task fitness under modified conditions, we tested all evolved MBs (the final generation) in four modified environment designs (see Fig 16(B)–16(E)). Generally, animats were allowed to inhabit the same location in the environment (albeit under penalty, see below), except in ***G***_***blocked***_ and ***G***_***blocked/no-penalty***_.

### Experiment design

We selected ***G***_***0*.*50***_ to be the baseline setup for evolution, to which we compared all other evolutionary setups. This was because ***G***_***0*.*50***_ showed the highest reliability ***R*** across group sizes. In sum, we came up with ***15*** different setups for the evolution of the animats (see Table 1). Using the MABE framework, we simulated each evolutionary setup ***30*** times. In each of these ***30*** evolutions, the evolutionary algorithm had ***10*,*000*** generations to converge on the final solution. A population of ***100*** genomes was mutated and evaluated in each generation. Each of these evaluations was repeated ***30*** times (30 “test runs”) with random starting positions, orientation, and selection order for simulating the animats movement serially. Random seeds were chosen using a Mersenne-Twister (mt19937) random number generator (see S2 Text for a more detailed explanation of the parameter sampling). After a genome was tested ***30*** times, it received a fitness score, which was computed based on the mean across the task performance of ***30*** single animats, with one being picked randomly from each of the 30 random test runs. In addition, in setup ***G***_***random***_ the group size varied for each of the 30 tests. The specific group size was drawn randomly from a vector (***[1*, *4*, *7*, *11*, *14*, *18*, *22*, *25*, *29*, *32*, *36*, *40*, *43*, *47*, *50*, *54*, *58*, *61*, *65*, *68*, *72]***). This vector simulates a uniform distribution between ***1*** and ***72***.

### The simulated life

The fitness function ***F*** that determines the probability of a genome being reproduced depends on two factors. First, animats ***A*** have to travel as often as possible through the gate (change the room, see Fig 16). Second, the animats need to avoid colliding with each other. Fischer et al. [18] already included the formal definitions of the fitness function as a weighted sum of the penalty for collision and the reward for crossing the gate (see Table 5 for the mathematical notation of Eqs 4 and 5):

$$f(a)=(\sum_{t=0}^{T-1}\{\begin{array}{l} 1,\;g(a,t,t+1)=1\;and\;g(a,t-100,\;t)=0\\ 0,otherwise \end{array})-(\sum_{t=0}^{T}\{\begin{array}{c} 0.075,\;c(x(a),y(a),t)>1\\ 0,\;otherwise \end{array}),$$ (4)

$$F(A)=\frac{\sum_{i=1}^{30}f(rand(A))}{30}.$$ (5)

**Table 5:** Mathematical notation as used in the fitness function *F(A)* and *f(a)*.

***a ∈ A***	A single animat ***a*** in the set of all animats ***A*** in a trial.
***f(a)***	The fitness of a single animat ***a***.
***F(A)***	The average fitness of all animats in ***A*** as clones of a single genome.
***rand(A)***	Picks a random animat ***a*** from the group ***A***.
***g(a*, *t***_***a***_**, *t***_***b***_***)***	Returns the number of gate-crossings between time ***t***_***a***_ and time ***t***_***b***_ for a single animat ***a***.
***t ∈ T***	A single time step ***t***, where ***t ∈ T*** and ***T = [1*, *2*, *…*, *499*, *500]***.
***c(x*,*y*, *t)***	Returns the number of animats at a specific position ***(x*,*y)*** at time ***t***.

The amount of reward (+***1*.*0*** *points*) is higher than the amount subtracted in the case of a penalty (-***0*.*075*** *points*). These numbers need to be chosen carefully. If the penalty is too low or the reward is too high, animats will keep moving from one room to the other through the gate (herding effect) and ignore the penalty. On the other hand, given a high penalty and low reward, animats will evolve hardly any movement. To further reduce the herding effect around the gate, there is a refractory period of ***100*** timesteps after receiving a reward before the same animat can receive another reward. Since each trial has a duration ***T*** of ***500*** timesteps, any one animat can receive a total fitness score of at most ***4*** *points* [18].

To investigate the coordination and cooperation of animats in groups, we let animats co-exist in the same environment (in contrast to previous studies in this scope [16,19,24]). Currently, we have not implemented co-evolution of animats with different genomes and have only evaluated a genome by generating animats as identical clones (with the same MBs). There was no active knowledge exchange (“communication”) between animats in this study. Animats had to develop the ability to distinguish which kind of sensory input to use for decision making. As specified above, sensors can only sense one position in front of–or on the side of (***G***_***3sides***_)–the animat and differentiate between static objects (walls) and dynamic objects (fellow animats), except for ***G***_***w = a***_.

Compared to the baseline setup, we included further evolutionary setups in which animats did not receive the collision penalty and/or were not able to overlap (***G***_***no-penalty***_, ***G***_***blocked*,**_ ***G***_***blocked/no-penalty***_). Those changes in the fitness function represented environmental rules which influenced the task difficulty. As a result, we were able to test the role that the imposed interaction conditions between animats played in order to achieve high task fitness under modified conditions.

### Post-evolutionary evaluation

#### Modified conditions

Post-evolutionary task fitness tests were designed as follows: First, we selected the ***30*** genomes of generation ***10*,*000*** (***10k***) for each of the ***15*** evolutionary setups (see Table 1). Second, each genome was tested across ***21*** conditions varying in group size in the *Original* test condition. To this end, we created groups of animat clones of the respective test group size for each of the 30*15 genomes. Test group sizes were uniformly distributed between ***1*** and ***72***. The interval of the relative group sizes is ***[1, 4, 7, 11, 14, 18, 22, 25, 29, 32, 36, 40, 43, 47, 50, 54, 58, 61, 65, 68, 72]***. A single animat is not a group, but we treat it as one in order to simplify notation.

In addition to varying group sizes in the baseline task design (*Original*), we created four modified test environments, as shown in Fig 16 (*Noisy Corners*, *Small Gate*, *4 Rooms*, *4 Messy Rooms*). Moreover, we included three additional test conditions in which we varied the interaction conditions of the animats (*No Penalty*, *Blocked*, *Blocked and no penalty*). Finally, we tested each of the ***30×15×21*** different configurations in each of the eight test environments.

For the statistical analysis and the main reliability evaluations, we defined a quantitative reliability measure ***R*** across group sizes in the *Original* environment design (see Eq 3 above). The modified test environments represented four independent samples of possible environmental modifications. For this reason, they were evaluated on their own in terms of the achieved task fitness ***TF***. The results of the remaining three test conditions with varying interaction properties mainly served to highlight differences between the evolutionary setups, rather than testing reliability per se.

#### Brain complexity

To evaluate the complexity of the evolved MBs, we employed two complimentary measures provided by integrated information theory (IIT) [19,46], ***Φ***^***Max***^ and the associated number concepts ***#Concepts(Φ***^***Max***^***)***. The core of IIT’s measures is an information theoretic, and probabilistic graph analysis [19] based on the state-to-state transition probabilities of the units, i.e., their update functions. Please refer to [19,20] for details on the evaluation. Very briefly, to evaluate the integrated information ***Φ*** (“big phi”) for a particular set of computational units *S* in state *S* = *s*, the first step is to assess which subsets *Y*⊆*S* specify positive integrated information *φ*>0 (“small phi”) within the system (the set’s “concepts”). *φ* captures how much a set of elements *Y* within the system in its state *y* constrains the prior and next states of other system subsets *V*_*t*±1_⊆*S*. In simplified terms:

$$\varphi (Y=y_{t})=\min_{t\pm 1}\left(\min_{\Psi }\left(D\left(\frac{\hat{p}(V_{t\pm 1}|y_{t})}{\Psi (\hat{p}(V_{t\pm 1}|y_{t}))}\right)\right)\right)$$ (6)

where Ψ partitions $\hat{p}(V_{t\pm 1}|y_{t})$ into the product distribution $\hat{p}(V_{1,t\pm 1}|y_{1,t})\times \hat{p}(V_{2,t\pm 1}|y_{2,t})$, and *D* is a distance measure between two probability distributions. The ^ (”hat”-symbol) above the probability function *p* indicates that probabilities are interventional (obtained from system perturbations) rather than observational [19,47]. *V*_*t*±1_ are chosen such that *φ*(*y*_*t*_) is maximal. Second, ***Φ*** is measured as the minimal difference that any system partition Ψ_S_ makes to the overall information specified by all subsets Y with φ(y_t_)>0. Again, in simplified terms:

$$\Phi =\underset{\Psi_{S}}{min}\left(D({\left\{\varphi (y_{t})\right\}}_{Y\subseteq S};\Psi_{S}({\left\{\varphi (y_{t})\right\}}_{Y\subseteq S}))\right)$$ (7)

For a given MB, we search across all sets of computational units *S* for the one with Φ^max^ = max_S_ Φ. ***Φ***^***Max***^ represents the highest possible integrated information the MB can achieve across all its subsets, which we used as an indicator for brain complexity [19].

All calculations were conducted using the IIT Python package *pyphi* [22], which we used in our work to calculate ***Φ***^***Max***^ and the corresponding number of concepts. Since the employed measures are state-dependent, we evaluated ***Φ***^***Max***^ and the number of concepts for every state a MB experienced during a lifetime (one trial) and selected the maximum value over all states as in [20]. S1 Fig in Supporting Information shows by way of example that it is essential for high ***Φ***^***Max***^ in a system that many elements are integrated, meaning also maintaining functional feedback loops within the system. In this study, we only considered the brain complexity of the final generation (***10k***) due to the computational complexity of calculations using *pyphi*.

#### Statistics

The evolved fitness values ***EF***, the reliability ***R***, and the IIT brain complexity measures were statistically evaluated across all evolutionary setups using a Kruskal-Wallis test, which showed a significant difference of the observed statistics between all groups taken together. Further, we used the Mann-Whitney-U test to evaluate the difference between pairs of evolutionary setups. Tables A-G in S1 Text lists all statistical tests that are a subject of discussion in the results and discussion section.

## Supporting information

S1 Fig Brain wiring diagram. **(a)**. Best animat in evolution *#4* under condition ***G***_***random***_ with an evolved fitness ***EF = 3*.*1*** and ***Φ***^***Max***^ ***= 0***. The network structure shows only few feedback loops, which cannot produce integrated information. **(b)** Best animat in evolution *#1* under condition ***G***_***random***_ with an evolved fitness ***EF = 2*.*9*** and ***Φ***^***Max***^ ***= 7*.*77***. The network structure shows much more connections, which integrated the network states and makes them interdependent.
(TIFF)

S1 Table MABE parameters. Parameters used to configure the Genetic Algorithm with in the MABE framework.
(DOCX)

S1 Text Statistical analysis. This file contains Tables A-G listing mean values and correlation coefficients of evaluated quantities, as well as the results of our Mann-Whitney-U Tests.
(DOCX)

S2 Text Parameter sampling. Description of the random seeds and random number generator used in this study.
(DOCX)

## References

[1] Spearman C. “General Intelligence,” Objectively Determined and Measured. Am J Psychol. 1904;15: 201–292.

[2] Gardner H. The theory of multiple intelligences. Ann Dyslexia. 1987;37: 19–35. 10.1007/BF02648057 24234985

[3] Garnier S, Gautrais J, Theraulaz G. The biological principles of swarm intelligence. Swarm Intell. 2007;1: 3–31. 10.1007/s11721-007-0004-y

[4] Pacala SW, Gordon DM, Godfray HCJ. Effects of social group size on information transfer and task allocation. Evol Ecol. 1996;10: 127–165. 10.1007/BF01241782

[5] Dorigo M, Trianni V, Şahin E, Groß R, Labella TH, Baldassarre G, et al Evolving Self-Organizing Behaviors for a Swarm-Bot. Auton Robots. 2004;17: 223–245. 10.1023/B:AURO.0000033973.24945.f3

[6] Weick KE, Sutcliffe KM, Obstfeld D. Organizing for High Reliability: Process of Collective Mindfulness. Cris Manag. 2008;3: 31–66. 10.1177/0020764009106599

[7] Weick KE, Roberts KH. Collective Mind in Organizations: Heedful Interrelating on Flight Decks. Adm Sci Q. 1993;38: 357 10.2307/2393372

[8] Oliver N, Senturk M, Calvard TS, Potocnik K, Tomasella M. Collective Mindfulness, Resilience and Team Performance. Acad Manag Proc. 2017;2017 10.5465/AMBPP.2017.12905abstract

[9] Fleck L. Genesis and Development of a Scientific Fact. 1983 10.2307/2067139

[10] Pinter-Wollman N, Penn A, Theraulaz G, Fiore SM. Interdisciplinary approaches for uncovering the impacts of architecture on collective behaviour. Philos Trans R Soc B Biol Sci. 2018;373 10.1098/rstb.2017.0232 29967298 PMC6030586

[11] Engel D, Malone TW. Integrated information as a metric for group interaction. Dovrolis C, editor. PLoS One. 2018;13 10.1371/journal.pone.0205335 30307973 PMC6181355

[12] List C, Philip Pettit. Group Agency and Supervenience. South J Philos. 2006;44: 1–22.

[13] Walsh J, Ungson GR. Organizational Memory. Acad Manag Rev. 1991;16: 57–91. 10.2307/258607

[14] Nonaka I. A firm as a knowledge-creating entity: a new perspective on the theory of the firm. Ind Corp Chang. 2000;9: 1–20. 10.1093/icc/9.1.1

[15] Tsoukas H. The firm as a distributed knowledge system: A constructionist approach. Strateg Manag J. 1996;17: 11–25. 10.1002/smj.4250171104

[16] Marstaller L, Hintze A, Adami C. The Evolution of Representation in Simple Cognitive Networks. Neural Comput. 2013;25: 2079–2107. 10.1162/NECO_a_00475 23663146

[17] Hintze A, Kirkpatrick D, Adami C. The structure of evolved representations across different substrates for artificial intelligence. 2018; Available: http://arxiv.org/abs/1804.01660

[18] Fischer D, Mostaghim S, Albantakis L. How swarm size during evolution impacts the behavior, generalizability, and brain complexity of animats performing a spatial navigation task. GECCO 2018 2018; 10.1145/3205455.3205646

[19] Oizumi M, Albantakis L, Tononi G. From the Phenomenology to the Mechanisms of Consciousness: Integrated Information Theory 3.0. Sporns O, editor. PLoS Comput Biol. 2014;10: 1–25. 10.1371/journal.pcbi.1003588 24811198 PMC4014402

[20] Albantakis L, Hintze A, Koch C, Adami C, Tononi G. Evolution of Integrated Causal Structures in Animats Exposed to Environments of Increasing Complexity. Polani D, editor. PLoS Comput Biol. 2014;10: e1003966 10.1371/journal.pcbi.1003966 25521484 PMC4270440

[21] König L, Mostaghim S, Schmeck H. Decentralized evolution of robotic behavior using finite state machines. Hettiarachchi S, editor. Int J Intell Comput Cybern. 2009;2: 695–723. 10.1108/17563780911005845

[22] Mayner WGP, Marshall W, Albantakis L, Findlay G, Marchman R, Tononi G. PyPhi: A toolbox for integrated information theory. Blackwell KT, editor. PLOS Comput Biol. 2018;14: e1006343 10.1371/journal.pcbi.1006343 30048445 PMC6080800

[23] Marshall W, Kim H, Walker SI, Tononi G, Albantakis L. How causal analysis can reveal autonomy in models of biological systems. Philos Trans R Soc A Math Phys Eng Sci. 2017;375: 20160358 10.1098/rsta.2016.0358 29133455 PMC5686412

[24] Edlund JA, Chaumont N, Hintze A, Koch C, Tononi G, Adami C. Integrated Information Increases with Fitness in the Evolution of Animats. Graham LJ, editor. PLoS Comput Biol. 2011;7: e1002236 10.1371/journal.pcbi.1002236 22028639 PMC3197648

[25] Marshall W, Gomez-Ramirez J, Tononi G. Integrated information and state differentiation. Front Psychol. 2016;7 10.3389/fpsyg.2016.00926 27445896 PMC4923128

[26] Miikkulainen R, Feasley E, Johnson L, Karpov I, Rajagopalan P, Rawal A, et al Multiagent Learning through Neuroevolution. Lecture Notes in Computer Science (including subseries Lecture Notes in Artificial Intelligence and Lecture Notes in Bioinformatics). 2012 pp. 24–46. 10.1007/978-3-642-30687-7_2

[27] Brown JL. Optimal group size in territorial animals. J Theor Biol. 1982;95: 793–810. 10.1016/0022-5193(82)90354-X

[28] Olson RS. Elucidating the Evolutionary Origins of Collective Animal Behavior. PhD Proposal. 2015.

[29] Olson RS, Hintze A, Dyer FC, Knoester DB, Adami C. Predator confusion is sufficient to evolve swarming behavior. J R Soc Interface. 2012;10: 20130305 10.1098/rsif.2013.0305 23740485 PMC4043163

[30] Olson RS, Knoester DB, Adami C. Critical interplay between density-dependent predation and evolution of the selfish herd. Proceeding fifteenth Annu Conf Genet Evol Comput Conf—GECCO ‘13. 2013; 247. 10.1145/2463372.2463394

[31] Karpov I V., Johnson LM, Miikkulainen R. Evaluating team behaviors constructed with human-guided machine learning. 2015 IEEE Conference on Computational Intelligence and Games (CIG). IEEE; 2015. pp. 292–298. 10.1109/CIG.2015.7317946

[32] Stanley KO, Cornelius R, Miikkulainen R, Silva TD, Gold A. Real-time Learning in the NERO Video Game. Proc First Artif Intell Interact Digit Entertain Conf. 2005;2003: 2003–2004.

[33] Stanley KO, Bryant BD, Miikkulainen R. Real-time neuroevolution in the NERO video game. IEEE Trans Evol Comput. 2005;9: 653–668. 10.1109/TEVC.2005.856210

[34] Hamann H. Evolution of Collective Behaviors by Minimizing Surprise. 14th Int Conf Synth Simul Living Syst (ALIFE 2014). 2014; 344–351. 10.1145/2739482.2768497

[35] Garnier S, Hamann H, Montes M, Christine DO, Eds TS, Hutchison D. Swarm Intelligence. In: Gerhard Goos, Hartmanis J, Leeuwen J van, editors. LNCS 8667 Brussels; 2014.

[36] Ishiwata H, Noman N, Iba H. Emergence of Cooperation in a Bio-inspired Multi-agent System. Lecture Notes in Computer Science (including subseries Lecture Notes in Artificial Intelligence and Lecture Notes in Bioinformatics). 2010 pp. 364–374. 10.1007/978-3-642-17432-2_37

[37] Reid CR, Lutz MJ, Powell S, Kao AB, Couzin ID, Garnier S. Army ants dynamically adjust living bridges in response to a cost-benefit trade-off. Proc Natl Acad Sci U S A. 2015;112: 15113–8. 10.1073/pnas.1512241112 26598673 PMC4679032

[38] Joshi NJ, Tononi G, Koch C. The Minimal Complexity of Adapting Agents Increases with Fitness. PLoS Comput Biol. 2013;9 10.1371/journal.pcbi.1003111 23874168 PMC3708884

[39] Sheneman L, Hintze A. Evolving autonomous learning in cognitive networks. Sci Rep. Springer US; 2017; 1–11. 10.1038/s41598-016-0028-x 29196623 PMC5711912

[40] Hintze A, Schossau J, Bohm C. The Evolutionary Buffet Method. Genetic Programming Theory and Practice XVI. 2019 pp. 17–36. 10.1007/978-3-030-04735-1_2

[41] Beer RD, Williams PL. Information Processing and Dynamics in Minimally Cognitive Agents. Cogn Sci. 2015;39: 1–38. 10.1111/cogs.12142 25039535

[42] Lizier JT, Prokopenko M, Zomaya AY. A Framework for the Local Information Dynamics of Distributed Computation in Complex Systems. 2014 pp. 115–158. 10.1007/978-3-642-53734-9_5

[43] Zenil H. Compression-based investigation of the dynamical properties of cellular automata and other systems. Arxiv Prepr arXiv09104042. 2009; 1–25. Available: http://arxiv.org/abs/0910.4042

[44] Clifford Bohm, Nitash C. G. AH. MABE (Modular Agent Based Evolver): A framework for digital evolution research. Proceedings of the European Conference on Artificial Life. MIT Press; 2017. pp. 76–83.

[45] Hintze A, Edlund JA, Olson RS, Knoester DB, Schossau J, Albantakis L, et al Markov Brains: A Technical Introduction. 2017; Available: http://arxiv.org/abs/1709.05601

[46] Tononi G. Integrated information theory. Scholarpedia. 2015;10: 4164 10.4249/scholarpedia.4164

[47] Ay N, Polani D. Information Flows in Causal Networks. Adv Complex Syst. 2008;11: 17–41. 10.1142/S0219525908001465

