# Supplementary figures and images for: How cognitive and environmental constraints influence the reliability of simulated animats in groups
Source: PLoS One. 2020 Feb 7;15(2):e0228879. doi: 10.1371/journal.pone.0228879 (PMC7006938; doi:10.1371/journal.pone.0228879)

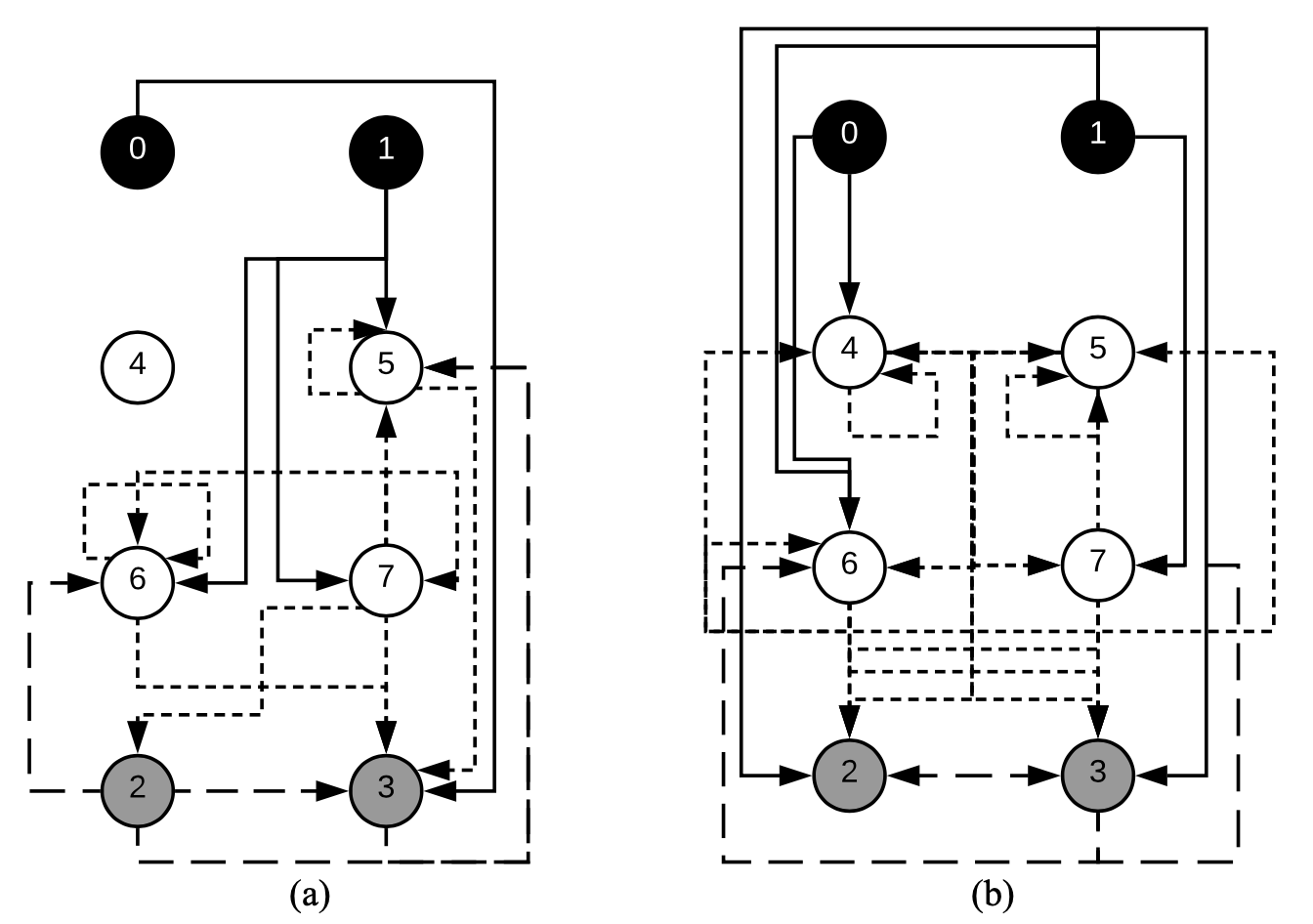

Supplement: S1 Fig — (a). Best animat in evolution #4 under condition Grandom with an evolved fitness EF = 3.1 and ΦMax = 0. The network structure shows only few feedback loops, which cannot produce integrated information. (b) Best animat in evolution #1 under condition Grandom with an evolved fitness EF = 2.9 and ΦMax = 7.77. The network structure shows much more connections, which integrated the network states and makes them interdependent. (TIFF) [file pone.0228879.s001.tiff]
