# Supplementary material for: How cognitive and environmental constraints influence the reliability of simulated animats in groups
Source: PLoS One. 2020 Feb 7;15(2):e0228879. doi: 10.1371/journal.pone.0228879 (PMC7006938; doi:10.1371/journal.pone.0228879)
Supplement: S1 Table — Parameters used to configure the Genetic Algorithm with in the MABE framework. (DOCX) [file pone.0228879.s002.docx]

**S1 Table.** Parameters used to configure the Genetic Algorithm with in the MABE framework.

| **Category** | **Setting** | **Value** |
| --- | --- | --- |
| **Genome setup** | Type | Circular |
|  | Alphabet Size | 256 |
|  | Sites Type | char |
|  | Initial Size | 5,000 |
|  | Mutation Point Rate | 0.005 |
|  | Mutation Copy/Delete Rate | 0.00002 |
|  | Minimal Mutation Copy/Delete Size | 128 |
|  | Maximum Mutation Copy/Delete Size | 512 |
|  | Minimal Size | 2,000 |
|  | Maximal Size | 20,000 |
| **Markov brain setup** | Type of Gates | Deterministic |
|  | Range of Inputs/Outputs per Gate | 1 to 4 |
| **Optimizer setup** | Type of Optimizer | Tournament |
|  | Tournament Size | 5 |
|  | Population Size | 100 |
|  | Elitism | No |
|  | Number of Parents | 1 (no crossover) |
