# Supplementary material for: How cognitive and environmental constraints influence the reliability of simulated animats in groups
Source: PLoS One. 2020 Feb 7;15(2):e0228879. doi: 10.1371/journal.pone.0228879 (PMC7006938; doi:10.1371/journal.pone.0228879)
Supplement: S1 Text — This file contains Tables A-G listing mean values and correlation coefficients of evaluated quantities, as well as the results of our Mann-Whitney-U Tests. (DOCX) [file pone.0228879.s003.docx]

S1 Text. Statistical Analysis.

This file contains Tables A-G listing mean values and correlation coefficients of evaluated quantities, as well as the results of our Mann-Whitney-U Tests.

**Table A.** Mean 〈…〉 and SEM values of the evolved fitness EF, reliability across group sizes in the original setup R, and brain complexity Φ^Max^ of the final evolved animats grouped by conditions. Roman numbers indicated the rank of the corresponding mean through all conditions. The results of brain complexity calculations for G_bigbrain_ are not available (NA) due to the computational complexity of the calculations.

| $\text{G}_{\text{i}}^{\text{10k}}$ | $\left\langle\boldsymbol{EF} \right\rangle$ | | SEM | $\left\langle\boldsymbol{R} \right\rangle$ | SEM | $\left\langle\boldsymbol{\phi}^{\boldsymbol{Max}} \right\rangle$ | SEM |
| --- | --- | --- | --- | --- | --- | --- | --- |
| 0.50 | (VIII) 3.1267 | 0.0727 | | (III) 2.9315 | 0.0845 | (III) 2.4921 | 0.2673 |
| random | (VI) 3.3051 | 0.0492 | | (II) 3.0257 | 0.0491 | (II) 2.6472 | 0.4319 |
| 1.00 | 2.4271 | 0.0822 | | (VIII) 2.3580 | 0.1415 | (VII) 1.8157 | 0.2595 |
| 0.75 | 2.6732 | 0.0891 | | (VII) 2.6239 | 0.1039 | (VI) 1.9209 | 0.3467 |
| 0.25 | (V) 3.3099 | 0.1188 | | (VI) 2.7311 | 0.0945 | (V) 2.1980 | 0.3278 |
| single | (II) 3.7356 | 0.0588 | | -2.2667 | 0.6666 | (X) 1.2476 | 0.1697 |
| bigbrain | (VII) 3.1847 | 0.1017 | | (IV) 2.7725 | 0.1047 | NA | NA |
| smallbrain | (X) 2.7296 | 0.0617 | | (IX) 2.3343 | 0.0809 | (IX) 1.1600 | 0.1906 |
| no-feedback | 1.9696 | 0.1131 | | (X) 1.7839 | 0.1165 | 0.3389 | 0.0824 |
| no-agent | 0.1788 | 0.1474 | | 0.3168 | 0.1865 | (VIII) 1.5529 | 0.3064 |
| 3sides | (III) 3.6092 | 0.1281 | | (I) 3.3808 | 0.1227 | 0.2483 | 0.0960 |
| w=a | 0.1793 | 0.0881 | | 0.2632 | 0.0868 | 0.6721 | 0.1025 |
| no-penalty | (I) 3.7867 | 0.0447 | | -2.8575 | 0.2115 | 0.9356 | 0.1348 |
| blocked/no-penalty | (IV) 3.3367 | 0.0532 | | 0.5958 | 0.2988 | (IV) 2.2055 | 0.2271 |
| blocked | (IX) 3.0994 | 0.0924 | | (V) 2.7442 | 0.0806 | (I) 3.0634 | 0.5598 |

**Table B**. The *p-values* of the Mann-Whitney-U Tests for the average mean of the evolved fitness score 〈EF〉 per condition. The p-value for the preceded Kruskal-Wallis-Test is 0.000.

| $\text{G}_{\text{i}}^{\text{10k}}$ ***/*** $\text{G}_{\text{i}}^{\text{10k}}$ | ***0.50*** | ***random*** | ***1.00*** | ***0.75*** | ***0.25*** | ***Single*** | ***bigbrain*** | ***smallbrain*** | ***no-feedback*** | ***no-agent*** | ***3sides*** | ***w=a*** | ***no-penalty*** | ***blocked/***  ***no-penalty*** |
| --- | --- | --- | --- | --- | --- | --- | --- | --- | --- | --- | --- | --- | --- | --- |
| ***random*** | 0.017 |  |  |  |  |  |  |  |  |  |  |  |  |  |
| ***1.00*** | 0.000 | 0.000 |  |  |  |  |  |  |  |  |  |  |  |  |
| ***0.75*** | 0.000 | 0.000 | 0.000 |  |  |  |  |  |  |  |  |  |  |  |
| ***0.25*** | 0.000 | 0.012 | 0.000 | 0.000 |  |  |  |  |  |  |  |  |  |  |
| ***single*** | 0.000 | 0.000 | 0.000 | 0.000 | 0.000 |  |  |  |  |  |  |  |  |  |
| ***bigbrain*** | 0.453 | 0.204 | 0.000 | 0.000 | 0.071 | 0.000 |  |  |  |  |  |  |  |  |
| ***smallbrain*** | 0.000 | 0.000 | 0.009 | 0.334 | 0.000 | 0.000 | 0.001 |  |  |  |  |  |  |  |
| ***no-feedback*** | 0.000 | 0.000 | 0.000 | 0.000 | 0.000 | 0.000 | 0.000 | 0.000 |  |  |  |  |  |  |
| ***no-agent*** | 0.000 | 0.000 | 0.000 | 0.000 | 0.000 | 0.000 | 0.000 | 0.000 | 0.000 |  |  |  |  |  |
| ***3sides*** | 0.000 | 0.001 | 0.000 | 0.000 | 0.007 | 0.087 | 0.001 | 0.000 | 0.000 | 0.000 |  |  |  |  |
| ***w=a*** | 0.000 | 0.000 | 0.000 | 0.000 | 0.000 | 0.000 | 0.000 | 0.000 | 0.000 | 0.112 | 0.000 |  |  |  |
| ***no-penalty*** | 0.000 | 0.000 | 0.000 | 0.000 | 0.000 | 0.344 | 0.000 | 0.000 | 0.000 | 0.000 | 0.017 | 0.000 |  |  |
| ***blocked/no-penalty*** | 0.004 | 0.208 | 0.000 | 0.000 | 0.068 | 0.000 | 0.098 | 0.000 | 0.000 | 0.000 | 0.001 | 0.000 | 0.000 |  |
| ***blocked*** | 0.435 | 0.048 | 0.000 | 0.000 | 0.001 | 0.000 | 0.372 | 0.000 | 0.000 | 0.000 | 0.000 | 0.000 | 0.000 | 0.012 |

**Table C**. The *p-values* of the Mann-Whitney-U Tests for the average brain complexity 〈Φ^Max^〉 per evolutionary setup. The results of brain complexity calculations for G_bigbrain_ are not available (NA) due to the computational complexity of the calculations. The p-value for the preceded Kruskal-Wallis-Test is 0.000.

| $\boldsymbol{G}_{\boldsymbol{i}}^{\boldsymbol{10}\boldsymbol{k}}$ ***/*** $\boldsymbol{G}_{\boldsymbol{i}}^{\boldsymbol{10}\boldsymbol{k}}$ | ***0.50*** | ***random*** | ***1.00*** | ***0.75*** | ***0.25*** | ***single*** | ***bigbrain*** | ***smallbrain*** | ***no-feedback*** | ***no-agent*** | ***3sides*** | ***w=a*** | ***no-penalty*** | ***blocked/***  ***no-penalty*** |  |
| --- | --- | --- | --- | --- | --- | --- | --- | --- | --- | --- | --- | --- | --- | --- | --- |
| ***random*** | 0.409 |  |  |  |  |  |  |  |  |  |  |  |  |  |  |
| ***1.00*** | 0.037 | 0.099 |  |  |  |  |  |  |  |  |  |  |  |  |  |
| ***0.75*** | 0.023 | 0.041 | 0.370 |  |  |  |  |  |  |  |  |  |  |  |  |
| ***0.25*** | 0.111 | 0.162 | 0.289 | 0.177 |  |  |  |  |  |  |  |  |  |  |  |
| ***single*** | 0.000 | 0.000 | 0.062 | 0.177 | 0.010 |  |  |  |  |  |  |  |  |  |  |
| ***bigbrain*** | NA | NA | NA | NA | NA | NA |  |  |  |  |  |  |  |  |  |
| ***smallbrain*** | 0.000 | 0.002 | 0.080 | 0.191 | 0.029 | 0.496 | NA |  |  |  |  |  |  |  |  |
| ***no-feedback*** | 0.000 | 0.000 | 0.000 | 0.000 | 0.000 | 0.000 | NA | 0.000 |  |  |  |  |  |  |  |
| ***no-agent*** | 0.002 | 0.007 | 0.124 | 0.259 | 0.049 | 0.389 | NA | 0.421 | 0.000 |  |  |  |  |  |  |
| ***3sides*** | 0.000 | 0.000 | 0.000 | 0.000 | 0.000 | 0.000 | NA | 0.000 | 0.092 | 0.000 |  |  |  |  |  |
| ***w=a*** | 0.000 | 0.000 | 0.000 | 0.001 | 0.000 | 0.001 | NA | 0.008 | 0.009 | 0.008 | 0.000 |  |  |  |  |
| ***no-penalty*** | 0.000 | 0.000 | 0.008 | 0.023 | 0.001 | 0.105 | NA | 0.154 | 0.000 | 0.138 | 0.000 | 0.057 |  |  |  |
| ***blocked/no-penalty*** | 0.196 | 0.375 | 0.134 | 0.063 | 0.331 | 0.001 | NA | 0.003 | 0.000 | 0.011 | 0.000 | 0.000 | 0.000 |  |  |
| ***blocked*** | 0.395 | 0.494 | 0.079 | 0.026 | 0.145 | 0.000 | NA | 0.002 | 0.000 | 0.006 | 0.000 | 0.000 | 0.000 | 0.337 |  |

**Table D.** The *p-values* of the Mann-Whitney-U Tests for the average number of concepts in the set of elements with Φ^Max^, according to IIT, per evolutionary setup. The results of brain complexity calculations for G_bigbrain_ are not available (NA) due to the computational complexity of the calculations. The p-value for the preceded Kruskal-Wallis-Test is 0.000.

| $G_{i}^{10k}$ / $G_{i}^{10k}$ | 0.50 | random | 1.00 | 0.75 | 0.25 | single | bigbrain | smallbrain | no-feedback | no-agent | 3sides | w=a | no-penalty | blocked/  no-penalty |
| --- | --- | --- | --- | --- | --- | --- | --- | --- | --- | --- | --- | --- | --- | --- |
| random | 0.372 |  |  |  |  |  |  |  |  |  |  |  |  |  |
| 1.00 | 0.246 | 0.381 |  |  |  |  |  |  |  |  |  |  |  |  |
| 0.75 | 0.070 | 0.120 | 0.272 |  |  |  |  |  |  |  |  |  |  |  |
| 0.25 | 0.265 | 0.339 | 0.465 | 0.239 |  |  |  |  |  |  |  |  |  |  |
| single | 0.007 | 0.014 | 0.083 | 0.221 | 0.051 |  |  |  |  |  |  |  |  |  |
| bigbrain | NA | NA | NA | NA | NA | NA |  |  |  |  |  |  |  |  |
| smallbrain | 0.000 | 0.000 | 0.010 | 0.019 | 0.002 | 0.044 | NA |  |  |  |  |  |  |  |
| no-feedback | 0.000 | 0.000 | 0.000 | 0.000 | 0.000 | 0.000 | NA | 0.000 |  |  |  |  |  |  |
| no-agent | 0.004 | 0.007 | 0.040 | 0.116 | 0.022 | 0.249 | NA | 0.187 | 0.000 |  |  |  |  |  |
| 3sides | 0.000 | 0.000 | 0.000 | 0.000 | 0.000 | 0.000 | NA | 0.000 | 0.059 | 0.000 |  |  |  |  |
| w=a | 0.000 | 0.000 | 0.000 | 0.000 | 0.000 | 0.000 | NA | 0.044 | 0.001 | 0.019 | 0.000 |  |  |  |
| no-penalty | 0.000 | 0.000 | 0.002 | 0.011 | 0.001 | 0.042 | NA | 0.462 | 0.000 | 0.193 | 0.000 | 0.038 |  |  |
| blocked/  no-penalty | 0.185 | 0.164 | 0.084 | 0.015 | 0.093 | 0.000 | NA | 0.000 | 0.000 | 0.000 | 0.000 | 0.000 | 0.000 |  |
| blocked | 0.418 | 0.430 | 0.300 | 0.142 | 0.350 | 0.025 | NA | 0.001 | 0.000 | 0.013 | 0.000 | 0.000 | 0.000 | 0.204 |

**Table E**. The *p-values* of the Mann-Whitney-U Tests for the average reliability score 〈R〉 per condition. The p-value for the preceded Kruskal-Wallis-Test is 0.000.

| $\boldsymbol{G}_{\boldsymbol{i}}^{\boldsymbol{10}\boldsymbol{k}}$ ***/*** $\boldsymbol{G}_{\boldsymbol{i}}^{\boldsymbol{10}\boldsymbol{k}}$ | ***0.50*** | ***random*** | ***1.00*** | ***0.75*** | ***0.25*** | ***Single*** | ***bigbrain*** | ***smallbrain*** | ***no-feedback*** | ***no-agent*** | ***3sides*** | ***w=a*** | ***no-penalty*** | ***blocked/***  ***no-penalty*** |
| --- | --- | --- | --- | --- | --- | --- | --- | --- | --- | --- | --- | --- | --- | --- |
| ***random*** | 0.023 |  |  |  |  |  |  |  |  |  |  |  |  |  |
| ***1.00*** | 0.000 | 0.000 |  |  |  |  |  |  |  |  |  |  |  |  |
| ***0.75*** | 0.001 | 0.000 | 0.260 |  |  |  |  |  |  |  |  |  |  |  |
| ***0.25*** | 0.198 | 0.002 | 0.003 | 0.008 |  |  |  |  |  |  |  |  |  |  |
| ***single*** | 0.000 | 0.000 | 0.000 | 0.000 | 0.000 |  |  |  |  |  |  |  |  |  |
| ***bigbrain*** | 0.450 | 0.077 | 0.001 | 0.003 | 0.210 | 0.000 |  |  |  |  |  |  |  |  |
| ***smallbrain*** | 0.000 | 0.000 | 0.206 | 0.036 | 0.001 | 0.000 | 0.001 |  |  |  |  |  |  |  |
| ***no-feedback*** | 0.000 | 0.000 | 0.000 | 0.000 | 0.000 | 0.000 | 0.000 | 0.000 |  |  |  |  |  |  |
| ***no-agent*** | 0.000 | 0.000 | 0.000 | 0.000 | 0.000 | 0.015 | 0.000 | 0.000 | 0.000 |  |  |  |  |  |
| ***3sides*** | 0.000 | 0.001 | 0.000 | 0.000 | 0.000 | 0.000 | 0.004 | 0.000 | 0.000 | 0.000 |  |  |  |  |
| ***w=a*** | 0.000 | 0.000 | 0.000 | 0.000 | 0.000 | 0.000 | 0.000 | 0.000 | 0.000 | 0.000 | 0.000 |  |  |  |
| ***no-penalty*** | 0.000 | 0.000 | 0.000 | 0.000 | 0.000 | 0.123 | 0.000 | 0.000 | 0.000 | 0.000 | 0.000 | 0.000 |  |  |
| ***blocked/no-penalty*** | 0.000 | 0.000 | 0.000 | 0.000 | 0.000 | 0.000 | 0.000 | 0.000 | 0.000 | 0.000 | 0.000 | 0.041 | 0.000 |  |
| ***blocked*** | 0.438 | 0.031 | 0.000 | 0.000 | 0.156 | 0.000 | 0.473 | 0.000 | 0.000 | 0.000 | 0.000 | 0.000 | 0.000 | 0.000 |

**Table F**. Spearman’s correlation coefficient ρ between the evolved fitness ***EF*** and brain complexity Φ^Max^ per condition (G_i_). Note that weak or non-significant correlation coefficients may be due to small variance in the final evolved fitness values in some conditions.

| $\text{EF}$ | $\Phi^{\mathrm{Max}}$ | | $\#Concepts(\Phi^{\mathrm{Max}})$ | |  |
| --- | --- | --- | --- | --- | --- |
|  | ρ | p-value | ρ | p-value |  |
| 0.5 | 0.6318 | 0.0002 | 0.6597 | 0.0001 |  |
| random | 0.2300 | 0.2215 | 0.0360 | 0.8504 |  |
| 1.00 | 0.4588 | 0.0108 | 0.6178 | 0.0003 |  |
| 0.75 | 0.2024 | 0.2834 | 0.1585 | 0.4028 |  |
| 0.25 | 0.6045 | 0.0004 | 0.4908 | 0.0059 |  |
| single | 0.1532 | 0.4189 | 0.3471 | 0.0602 |  |
| bigbrain | NA | NA | NA | NA |  |
| smallbrain | -0.4112 | 0.1011 | -0.2983 | 0.2449 |  |
| no-feedback | -0.1034 | 0.5936 | -0.1034 | 0.5936 |  |
| no-agent | 0.0439 | 0.8212 | 0.0768 | 0.6921 |  |
| 3sides | 0.2847 | 0.1273 | 0.2804 | 0.1333 |  |
| w=a | -0.0985 | 0.6047 | 0.0430 | 0.8214 |  |
| no-penalty | -0.1293 | 0.4960 | -0.1067 | 0.5748 |  |
| blocked/no-penalty | 0.2598 | 0.1656 | 0.4160 | 0.0222 |  |
| blocked | 0.4626 | 0.0100 | 0.6275 | 0.0002 |  |

**Table G.** Task fitness values of all conditions ***G_i_*** in the five evaluated environments.

| $\left\langle\left( TF \right)_{m} \right\rangle^{GS}$ | **Map *m =*** | | | | |
| --- | --- | --- | --- | --- | --- |
|  | *Original* | *Noisy Corners* | *Small Gate* | *4 Rooms* | *4 Messy Rooms* |
| ***0.25*** | 2.71 | 0.98 | 1.50 | 1.54 | 0.80 |
| ***random*** | 3.02 | 0.94 | 1.60 | 1.50 | 0.70 |
| ***0.50*** | 2.89 | 0.84 | 1.83 | 1.66 | 0.87 |
| ***0.75*** | 2.66 | 1.08 | 1.86 | 1.94 | 1.06 |
| ***1.00*** | 2.48 | 0.96 | 1.78 | 1.82 | 1.13 |
| ***single*** | -3.59 | -4.22 | -5.53 | -4.08 | -5.80 |
| ***bigbrain*** | 2.96 | 0.78 | 1.32 | 1.31 | 0.64 |
| ***smallbrain*** | 2.54 | 0.44 | 2.09 | 2.47 | 1.46 |
| ***no-feedback*** | 1.94 | 0.64 | 1.58 | 2.20 | 1.18 |
| ***no-agent*** | -1.61 | -2.18 | -2.75 | -2.68 | -4.29 |
| ***3sides*** | 3.33 | 0.64 | 2.56 | 3.13 | 1.59 |
| ***w=a*** | 0.13 | -0.11 | -0.45 | -0.85 | -2.36 |
| ***no-penalty**** | 3.75 [-3.88] | 2.83 | 1.95 | 0.94 | 0.31 |
| ***blocked/no-penalty**** | 3.08 [-0.11] | 0.61 | 1.44 | 1.74 | 0.75 |
| ***blocked**** | 2.87 [2.88] | 0.75 | 1.83 | 1.61 | 0.93 |

* The condition was tested in the environment with the interaction parameters they evolved in. The corresponding values of ***R*** (evaluated in the *Original* environment under standard settings: active penalty, blocking disabled) are added in parenthesis.
