# Supplementary material for: How cognitive and environmental constraints influence the reliability of simulated animats in groups
Source: PLoS One. 2020 Feb 7;15(2):e0228879. doi: 10.1371/journal.pone.0228879 (PMC7006938; doi:10.1371/journal.pone.0228879)
Supplement: S2 Text — Description of the random seeds and random number generator used in this study. (DOCX) [file pone.0228879.s004.docx]

S2 Text. Parameter sampling

Description of the random seeds and random number generator used in this study.


We used a Mersenne-Twister (*mt19937*) random number generator throughout the simulation. Each experiment condition ***G_i_*** was evolved ***30*** times on ***30*** distinct random seeds (corresponding to the ***N*** = 30 different evolution simulations per evolutionary setup ***G_i_***). The set of ***30*** distinct random seeds was manually chosen to make the experiments reproducible. The random number generator was used to draw a set of ***30*** unique samples for the starting positions as each genome was evaluated ***30*** times per generation. In addition, the random number generator was used to draw ***30×500*** samples per seed for the selection order for each simulation trial, which determines the order in which the individual animats updated their position and orientation in each of the ***500*** time steps per seed. In other words, the animat’s perception and reaction was processed serially under a random sequence. Note that the update sequence per seed remained the same across all ***10,000*** generations per simulation trial, while the distribution of the starting orientation (up, down, left, right) of the animats was drawn continuously, differing for each evaluation. All numbers were drawn from a uniform distribution. To reduce biases produced by the random number generator, we performed the post-evolutionary tests on different machines and random seeds. This means that initial conditions differed between the evolution simulations and the post-evolutionary tests.
